# Consensus Pharmacophore Strategy For Identifying Novel SARS-Cov-2 M^pro^ Inhibitors from Large Chemical Libraries

**DOI:** 10.1021/acs.jcim.3c01439

**Published:** 2024-03-12

**Authors:** Angel J. Ruiz-Moreno, Raziel Cedillo-González, Luis Cordova-Bahena, Zhiqiang An, José L. Medina-Franco, Marco A. Velasco-Velázquez

**Affiliations:** † School of Medicine, Universidad Nacional Autónoma de México, Mexico City 04510, Mexico; ‡ Graduate Program in Biochemical Sciences, Universidad Nacional Autónoma de México, Mexico City 04510, Mexico; § DIFACQUIM Research Group, School of Chemistry, Universidad Nacional Autónoma de México, Mexico City 04510, Mexico; ∥ Consejo Nacional de Humanidades, Ciencias y Tecnología, Mexico City 03940, Mexico; ⊥ Texas Therapeutics Institute, Brown Foundation Institute of Molecular Medicine, University of Texas Health Science Center, Houston, Texas 77030, United States

## Abstract

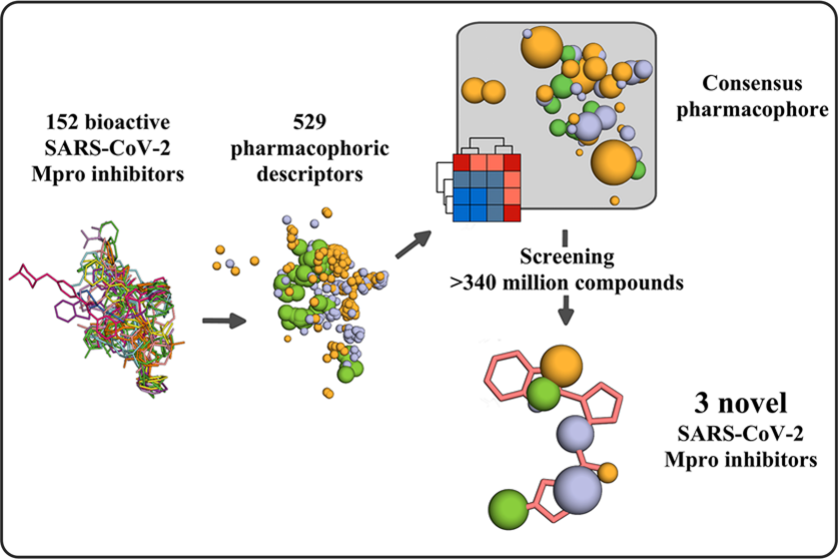

The severe acute respiratory syndrome coronavirus 2 (SARS-CoV-2) main Protease (M^pro^) is an enzyme that cleaves viral polyproteins translated from the viral genome and is critical for viral replication. M^pro^ is a target for anti-SARS-CoV-2 drug development, and multiple M^pro^ crystals complexed with competitive inhibitors have been reported. In this study, we aimed to develop an M^pro^ consensus pharmacophore as a tool to expand the search for inhibitors. We generated a consensus model by aligning and summarizing pharmacophoric points from 152 bioactive conformers of SARS-CoV-2 M^pro^ inhibitors. Validation against a library of conformers from a subset of ligands showed that our model retrieved poses that reproduced the crystal-binding mode in 77% of the cases. Using models derived from a consensus pharmacophore, we screened >340 million compounds. Pharmacophore-matching and chemoinformatics analyses identified new potential M^pro^ inhibitors. The candidate compounds were chemically dissimilar to the reference set, and among them, demonstrating the relevance of our model. We evaluated the effect of 16 candidates on M^pro^ enzymatic activity finding that seven have inhibitory activity. Three compounds (1, 4, and 5) had IC_50_ values in the midmicromolar range. The M^pro^ consensus pharmacophore reported herein can be used to identify compounds with improved activity and novel chemical scaffolds against M^pro^. The method developed for its generation is provided as an open-access code (https://github.com/AngelRuizMoreno/ConcensusPharmacophore) and can be applied to other pharmacological targets.

## 1 Introduction

Severe acute respiratory syndrome coronavirus 2 (SARS-CoV-2), identified in December 2019, is an enveloped, nonsegmented, positive-sense RNA coronavirus (CoV) responsible for the COVID-19 pandemic. This pandemic has profoundly impacted global health, the economy, and daily life.^1−3^ The genetic code of SARS-CoV-2 shares more than 89% similarity with the SARS-CoV genes.^4−6^ Infection by SARS-CoV-2 leads to the production of pp1a and pp1ab polypeptides from the viral genome, which are essential for viral replication.^7−9^ These polypeptides undergo proteolytic self-cleavage to produce 11 and 16 distinct nonstructural proteins (NSPs), respectively. SARS-CoV-2 encodes two proteases essential for this process: papain-like protease (PLpro) and main protease (M^pro^).^10^ The high level of evolutionary conservation of M^pro^ across CoV indicates that M^pro^ (also known as 3C-like protease; 3CLpro) is crucial for virus replication.^11^

The M^pro^ structure consists of three domains: I and II, made of antiparallel β-sheets, and III, made of α-helices. Its active site, located between domains I and II, is formed by four conserved subpockets (S1, S1′, S2, and S4) that facilitate substrate interactions.^12^ Subpocket S1′, contains the catalytic dyad His41-Cys145, is crucial for substrate anchoring via a covalent thioester bond to Cys145.^13^ The substrate binding mode is stabilized by hydrogen bonds in S1, noncovalent interactions with S1, S2, and S4, and hydrophobic interactions with S2 and S3.^14,15^ M^pro^ cleaves the viral polyproteins at 11 sites, releasing NSPs involved in RNA replication and transcription.^16^ Inhibiting M^pro^ disrupts this process by reducing the production of functional proteins essential for viral replication and assembly, making M^pro^ a key target for antiviral drug discovery and development against SARS-CoV-2 and other coronaviruses.^17,2,18^

Recent studies have discovered various M^pro^ inhibitors,^19−22^ such as ensitrelvir (S-217622), a nonpeptidic inhibitor developed through virtual screening and drug design,^23^ and Pfizer’s nirmatrelvir, which effectively treats COVID-19 when used with ritonavir.^24,25^ Other M^pro^ inhibitors have been discovered using molecular docking,^26−29^ molecular dynamics,^30−32^ QSAR,^33−35^ ligand-based design,^36−39^ and pharmacophore-matching.^40−43^

Starting from numerous M^pro^ crystals with inhibitors bound to the active site of the M^pro^ catalytic domain from Protein Data Bank (PDB),^44^ we produced a consensus pharmacophore to identify potential SARS-CoV-2 M^pro^ ligands from a large and diverse section of the chemical universe. This method overlaid bioactive conformers in the M^pro^ catalytic cavity, reducing pharmacophoric descriptors by grouping them based on interaction type, spatial position, and frequency of appearance. When tested against a library of conformers, our consensus pharmacophore correctly reproduced the crystallographic binding pose for 77% of compounds in the validation set. Screening over 340 million compounds identified 72 new potential M^pro^ inhibitors with chemical diversity, demonstrating the relevance and applicability of our model. As a proof of concept, we evaluated the effect of 16 compounds and found that seven inhibited M^pro^ enzymatic activity. Compounds 1, 4, and 5 were experimentally validated as M^pro^ inhibitors, displaying IC_50_ values in the midmicromolar range.

## 2 Materials and Methods

### 2.1 Ligand Selection and Pharmacophore Modeling

The crystallographic structures of M^pro^ were obtained from the UniProt REST API using the access code P0DTC1 for the Replicase Polyprotein 1a of SARS-CoV (March 2021, Figure S1).^45^ The generation of the consensus pharmacophore model utilized the Consensus Pharmacophore Python library, which can be found at https://github.com/AngelRuizMoreno/ConsensusPharmacophore. This library has two main modules: *Structures* and *Pharmacophores*. The module *Structures* searches for proteins and related information, including crystallographic structures, using UniProt’s REST API. It can also perform structural alignments for ligand-protein complexes and extract ligand or ligand–receptor pharmacophores using Pharmit binary.^46^ Conversely, the *Pharmacophores* module extracts pharmacophoric information from the Pharmer/Pharmit models. It facilitates the generation of consensus pharmacophores while also providing tools for visualizing pharmacophoric descriptors and exporting the results in Pymol^47^ and JSON formats, allowing for further manipulation, visualization, and pharmacophore matching for virtual screening approaches.

For this study, the Pharmit binary (latest 11–03–2016)^46^ was employed to generate individual ligand–receptor pharmacophore models for 152 aligned M^pro^ complexes. The resulting pharmacophoric descriptors were then classified according to their physicochemical characteristics including hydrogen bond donors, acceptors, and hydrophobic elements. These descriptors were then clustered based on their spatial location, and the center of mass for each cluster was determined. This step was critical in determining a consensus point for each cluster, considering the frequency of the occurrence of each point within it. Additionally, the radius of each point was determined by considering the dispersion of descriptors within the cluster, thus allowing for the weighted position, size, and frequency of a group of descriptors sharing identical physicochemical properties to be considered as a single consensus pharmacophoric point.

Hierarchical clustering, employing a complete linkage algorithm, was utilized to generate consensus pharmacophoric descriptors from 152 pharmacophoric models. A threshold set to 0.17 times the maximum distance between clusters was chosen for this purpose, which accurately captured the diversity inherent in multiple ligand–receptor pharmacophoric models. Clusters were formed when points were within 1.5 Å of each other. This distance criterion was selected to approximate the spacing of hydrogen bond donor or acceptor functionalized carbons, allowing for the independent characterization of atoms interacting with the receptor.^48−50^

### 2.2 Consensus Pharmacophore Validation

A set of 78 cocrystallized ligands, selected from a reference dataset, was used to validate the consensus pharmacophore. The selection criteria for these ligands were aimed at ensuring a broad representation of chemical space and included: (i) chemical diversity, with a similarity threshold set at ≤0.5 to avoid redundancy; (ii) a molecular mass range from 200 to 700 g/mol, to include a wide variety of sizes; (iii) a limit of up to 17 rotatable bonds, to maintain a manageable degree of conformational flexibility; and (iv) the presence of at least three pharmacophoric features, to ensure that the ligands had sufficient complexity for meaningful pharmacophore matching.

A conformer library was generated by using the RDKit ETKDG v2 algorithm. This approach produced diverse, energetically favorable conformations for each ligand, with a root-mean-square deviation (RMSD) cutoff of ≥0.5 Å to ensure conformational diversity. Molecules with fewer rotatable bonds produced approximately 100 conformers, whereas those with greater flexibility produced up to 250 nonredundant conformers. This variation in the number of conformers generated reflects the trade-off between the need for thorough sampling of the conformational space and computational efficiency. The conformer library was then subjected to pharmacophore matching by using Pharmit. This process entailed selecting consensus points that matched the reference pharmacophoric model of each ligand within the validation set. The matching process aimed to find conformers that closely resembled the pharmacophoric arrangement of features in the reference ligands. A successful match was considered as an RMSD less than 2.5 Å between the best matching conformer and the original reference ligand. This validation method not only tests the accuracy of the consensus pharmacophoric model in reproducing known ligand conformations but also shows how it can be used to identify potential inhibitors based on pharmacophore matching. This validation process is critical for demonstrating the utility of the consensus pharmacophore model in drug discovery efforts, particularly in the identification and optimization of novel inhibitors that target specific proteins or enzymes.

### 2.3 High-Throughput Virtual Screening

Based on the consensus pharmacophore, we generated new models, termed “submodels,″ which contained seven to eight pharmacophoric descriptors. The points for each submodel were chosen based on their frequency, as indicated by weight and center of mass calculations, as well as their physicochemical diversity. To ensure interaction with the M^pro^ catalytic residues His41 and Cys145, descriptors within the same category were kept at a minimum distance of 1.5 Å.

For pharmacophoric matching, we employed the extensive libraries from Pharmit, which include^46^ ChEMBL,^51^ ChemDiv,^52^ ChemSpace,^53^ MCULE,^54^ MCULE-ULTIMATE,^55^ MolPort,^56^ NCI Open Chemical Repository,^57^ PubChem,^58^ LabNetwork,^59^ and ZINC.^60^ This comprehensive screening encompassed a total of 343,353,042 chemical entities.

To prepare the Pharmit libraries, canonical SMILES were deduplicated, molecules were protonated at pH 7.4 via OpenBabel^61^ using the default parameters, resulting in a main tautomer for each compound. Also, salts were removed, leaving only the largest molecule component. Subsequently, up to ten conformers per molecule were generated using the Universal Force Field (UFF)^62^ via RDKit. The pharmacophore-matching compounds underwent local optimization within the M^pro^ catalytic site using SMINA (latest 15–10–2019),^63^ an Autodock Vina fork optimized for energetic convergence. This approach facilitates the rapid prediction of a predominant binding mode of ligands to a protein without the need for global optimization, thereby increasing computational efficiency.

For these analyses, the M^pro^ structure (PDB ID: 6M2N) was prepared by adding polar hydrogen atoms, optimizing hydrogen bonds, assigning atomic charges, and removing atomic clashes. The RMSD between poses pre- and postlocal optimization as well as ligand efficiency (LE), which is calculated as the ratio of SMINA pose scoring to the number of heavy atoms in the ligand, were used to evaluate candidate compounds. This methodology allowed us to use detailed pharmacophore modeling to identify potential inhibitors.

### 2.4 Similarity Analysis

To refine our selection of potential inhibitors and better understand the chemical space covered by our candidates, we compared the structural similarity of the reference compounds to those of the screened candidates.

For molecular fingerprinting, we used the Extended Connectivity Fingerprint (ECFP4). ECFP4 is especially good at capturing molecular topology by considering atoms and their connectivity up to four bonds away.^64^ Later, hierarchical clustering based on the Tanimoto coefficient^65^ was conducted to group the compounds according to their similarity to each other.

### 2.5 Compounds

Compounds 1–16 were purchased from Enamine-REAL (https://enamine.net/compound-collections/real-compounds/real-database, October 12th, 2023) and dissolved to 10 mM in DMSO for analysis. The compounds are as follows:

*Compound 1*, 7-fluoro-N-[4-methyl-2-(2,2,2-trifluoroethoxy)phenyl]-2-oxo-1,2,3,4-tetrahydroquinoline-4-carboxamide (*cat. ID Z812112836*). *Compound 2*, N-[4-methyl-2-(2,2,2-trifluoroethoxy)phenyl]-2-oxo-1,2,3,4-tetrahydroquinoline-4-carboxamide (*cat. ID Z818127562*). *Compound 3*, 7-fluoro-N-(4-fluoro-2-propoxyphenyl)-2-oxo-1,2,3,4-tetrahydroquinoline-4-carboxamide (*cat. ID Z1417871928*). *Compound 4*, N-(2-ethoxy-4-methylphenyl)-2-oxo-1,2,3,4-tetrahydroquinoline-4-carboxamide *(cat. ID Z738201348*). *Compound 5*, N-[2-(1H-1,3-benzodiazol-2-yl)phenyl]-5-cyclopropyl-1H-pyrazole-3-carboxamide (*cat. ID Z1175261943*). *Compound 6*, N-[3-(cyclohexylmethoxy)pyridin-2-yl]-2-oxo-1,2,3,4-tetrahydroquinoline-4-carboxamide (*cat. ID Z1038519764*). *Compound 7*, N-(3-carbamoylcyclobutyl)-5-chloro-2-(1H-1,2,3,4-tetrazol-1-yl)benzamide (*cat. ID Z3336817301*). *Compound 8*, 5-bromo-N-(3-carbamoylcyclobutyl)-2-(1H-1,2,3,4-tetrazol-1-yl)benzamide (*cat. ID Z8046404987*). *Compound 9*, 3-ethyl-N-[2-(3-ethyl-1H-1,2,4-triazol-5-yl)phenyl]-1H-pyrazole-5-carboxamide (*cat. ID Z1518691661*). *Compound 10*, N-[1-(3-ethoxypropyl)-1H-1,3-benzodiazol-2-yl]-2-oxopiperidine-4-carboxamide (*cat. ID Z1420032055*). *Compound 11*, methyl 4-ethyl-1-(2-oxopiperidine-4-amido)cyclohexane-1-carboxylate (*cat. ID Z1420721434*). *Compound 12*, N-[2-(1H-1,3-benzodiazol-2-yl)phenyl]-3-(trifluoromethyl)-1H-pyrazole-5-carboxamide (*cat. ID Z2027589711*). *Compound 13*, N-(4-bromo-2-ethoxyphenyl)morpholine-2-carboxamide (*cat. ID Z2450025896*). *Compound 14*, 2-{[(4-bromo-2-ethoxyphenyl)carbamoyl]amino}propanamide (*cat. ID Z1434212207*). *Compound 15*, 2-[(2-hydroxy-3-methoxy-5-methylphenyl)formamido]acetamide (*cat. ID Z1228665631*). *Compound 16*, N-(4-chloro-2-methoxy-5-methylphenyl)-2-oxopiperidine-4-carboxamide (*cat. ID Z1418979185*).

### 2.6 M^pro^ Enzymatic Activity Assays

The evaluation of M^pro^ enzymatic activity was conducted using a continuous kinetic fluorescence resonance energy transfer (FRET) assay according to the protocol and specifications provided by Reaction Biology Corp. (Malvern, PA, USA). Briefly, candidate compounds were incubated with recombinant M^pro^ in a reaction buffer composed of 25 mM Tris (pH 7.3), 1 mM EDTA, and 0.005% Triton X-100. Since DTT interferes with the efficacy of certain inhibitors,^66^ we performed FRET assays in the absence of DTT, as previously suggested for the initial screenings of potential M^pro^ inhibitors.^67,68^ The compounds were tested at a fixed concentration of 100 μM (all compounds) or across nine concentrations using a 3-fold serial dilution starting at 100 μM (compounds 1, 4, and 5). The protease activity was continuously monitored by measuring the increase in the fluorescence signal (excitation at 340 nm and emission at 492 nm), following the addition of a fluorogenic substrate [NH2-C(EDANS)VNSTQSGLRK(DABCYL)M-COOH]. This substrate is designed to emit fluorescence when cleaved by M^pro^, acting as a direct indicator of enzymatic activity. The initial rates of enzyme activity were calculated using linear regression analysis of the early linear portion of the kinetic curve. Normalization of activity was performed using controls consisting of the reaction buffer without the compound (vehicle control) and without the enzyme (baseline control), which represented the maximal and minimal responses, respectively. GC376, a reported M^pro^ inhibitor,^69^ was used as a positive control. Single-concentration assays were performed on two independent occasions, whereas dose–response curves were performed twice for compound 1 and once for compounds 4 and 5. The dose–response curves and half-maximal inhibitory concentration (IC_50_) values were calculated using Prism10 v 10.1.1 (GraphPad) software using the built-in equation “concentration of inhibitor vs normalized response-variable slope”.

### 2.7 Molecular Dynamics Simulations

The molecular dynamics (MD) simulations for the dimeric form of M^pro^-ligand complexes were conducted using the GROMACS 2021.6^70^ software suite, applying the CHARMM36m force field. Ligand parameters were defined utilizing the CHARMM GUI^71^ ensuring that the chemical and physical properties of the ligand were accurately represented within the force field. For each complex, a 99 Å periodic cubic simulation box was built for each complex to minimize the boundary effects. This box was then filled with water molecules using the TIP3P water model. All histidine residues in the protein were protonated to reflect their protonation states at pH of 7.4. To ensure electrical neutrality of the system, sodium (Na^+^) and chloride (Cl^–^) ions were added, resulting in an ionic strength of 0.15 M. The system underwent an initial energy minimization using the steepest descent algorithm to rapidly reduce any steric clashes or high-energy configurations. This was followed by equilibration under constant volume and temperature (NVT ensemble) conditions using a modified Berendsen thermostat to set the system temperature at 310.15 K. The LINCS algorithm was used to constrain bond lengths involving hydrogen atoms, allowing for a 2 fs integration time step during the simulation. Finally, simulations were run at a constant pressure of 1 bar and a temperature of 310.15 K for 250 ns with system trajectories saved every 10 ps. MD analysis was conducted by using the gmx_rms tools within the GROMACS suite. The analysis involved calculating the RMSD using heavy atoms from the ligands and the alpha-carbon atoms of Mpro protomers. Additionally, RMSF measurements were obtained for the alpha-carbon atoms of the M^pro^ protomers. All MD simulations and analyses were performed in triplicate.

### 2.8 Binding Free Energy

The binding free energy of each M^pro^-ligand complex was calculated employing the Molecular Mechanics/Poisson–Boltzmann Surface Area (MM/PBSA) and Molecular Mechanics/Generalized Born Surface Area (MM/GBSA) methods.^72^ To ensure a thorough and representative analysis, snapshots (frames) were selected at intervals of 100 ps from the final 50 ns of the MD simulations. This strategy allows a comprehensive exploration of the bioactive conformations of the ligand throughout the simulation, capturing a wide range of interactions and conformations that the ligand may adopt when bound to M^pro^.

## 3 Results and Discussion

### 3.1 Consensus Pharmacophore Generation

**Figure 1:**
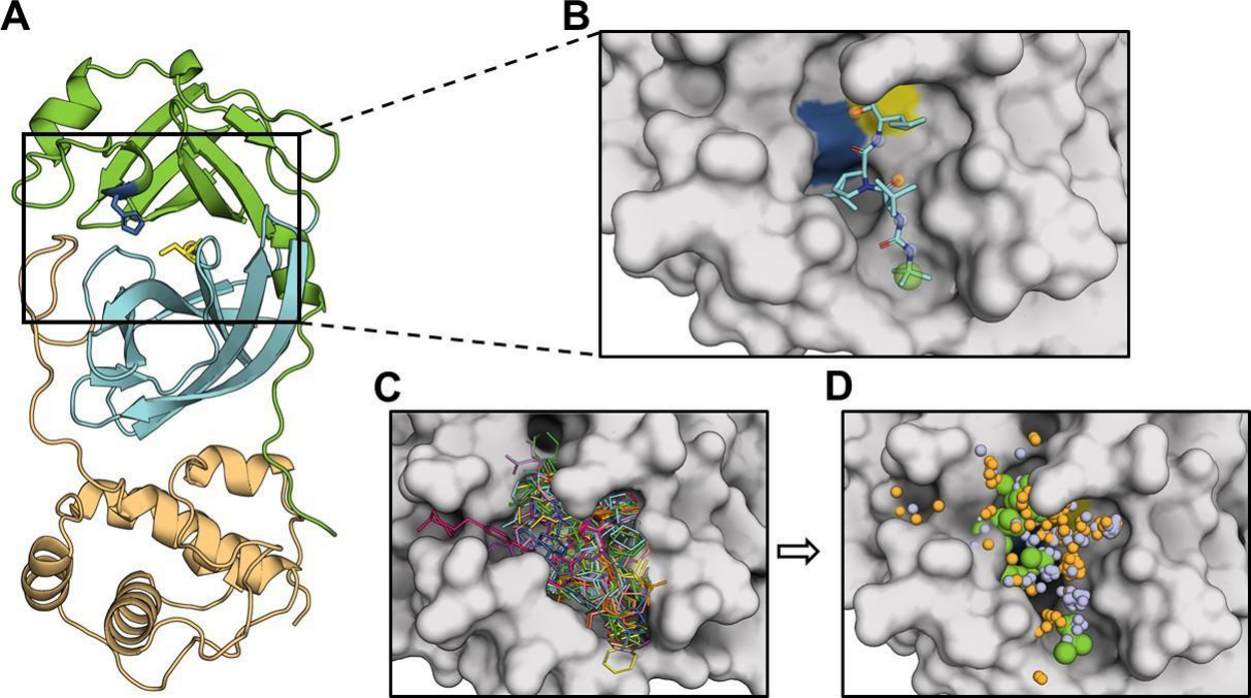
Cocrystallized ligands of M^pro^and experimental-derived pharmacophoric descriptors. (A) Structure of M^pro^ represented in ribbons and colored by domain. The catalytic residues His41 and Cys145 are shown as sticks and colored blue and yellow, respectively. (B) Surface representation of M^pro^ catalytic cavity with a bioactive ligand (PDB ID: 6M2N) showing the pharmacophoric descriptors extracted by using Pharmer. (C) Overlay of the 152 distinct ligands analyzed in this study. (D) Spatial position of all identified pharmacophoric descriptors within the M^pro^ catalytic cavity. Pharmacophoric descriptors are represented as spheres: hydrophobic: green; hydrogen bond acceptors: orange; hydrogen bond donors: purple.

We retrieved a comprehensive dataset of 177 M^pro^ crystals that met the criteria outlined in the methods (Table S1A–C). Pharmacophoric modeling was applied to these 177 structures, which included 152 different ligands interacting with the catalytic domain (Figure 1A,B). These ligands cover the critical subpockets (S1, S1′, S2, and S4) of M^pro^, demonstrating the diversity and comprehensiveness of the dataset in capturing the interaction landscape within the catalytic cavity (Figure 1C). We identified 529 pharmacophoric descriptors (Table S2A) from the ligands across the M^pro^ catalytic site, enabling a detailed mapping of the interaction potential within this critical region (Figure 1D).

**Figure 2:**
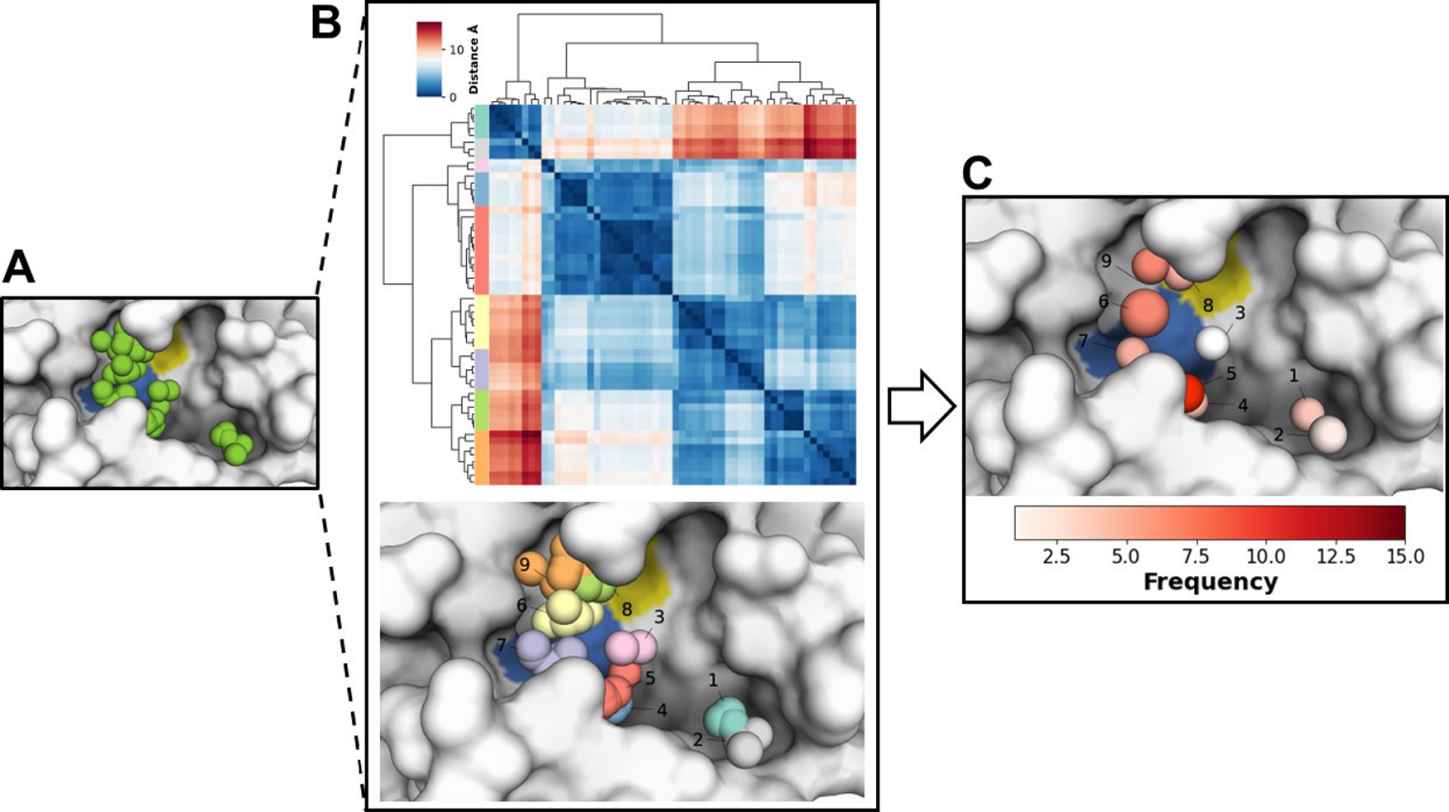
Example of clustering and identification of consensus pharmacophoric points. (A) Spatial position of 56 hydrophobic descriptors, identified from all 152 ligands analyzed, in the M^pro^ catalytic cavity. (B) Hierarchical clustering of spatial positions for hydrophobic descriptors in nine different clusters (upper part). Individual hydrophobic descriptors within the catalytic pocket are colored by its cluster (lower part). (C) Consensus hydrophobic points with spatial position defined by the center of mass, their radius, and cluster size (frequency) of each point. The surfaces of the catalytic residues His41 and Cys145 are colored in blue and yellow, respectively.

Pharmacophoric descriptors from all ligands were classified as hydrophobic (Figure 2A), hydrogen bond acceptors (Figure S1A), and hydrogen bond donors (Figure S2A) based on their physicochemical properties. The descriptors in each group were then clustered according to their spatial distances (Figures 2B, S1B and S2B), resulting in a manageable and interpretable set of consensus points. These points, characterized by their center of mass and radius, represent the aggregated interaction potential derived from the dataset, thereby providing a multidimensional view of the pharmacophore landscape. Furthermore, information about cluster size was included in the points to identify descriptors with a higher frequency (Figures 2C, S1C and S2C).

**Figure 3:**
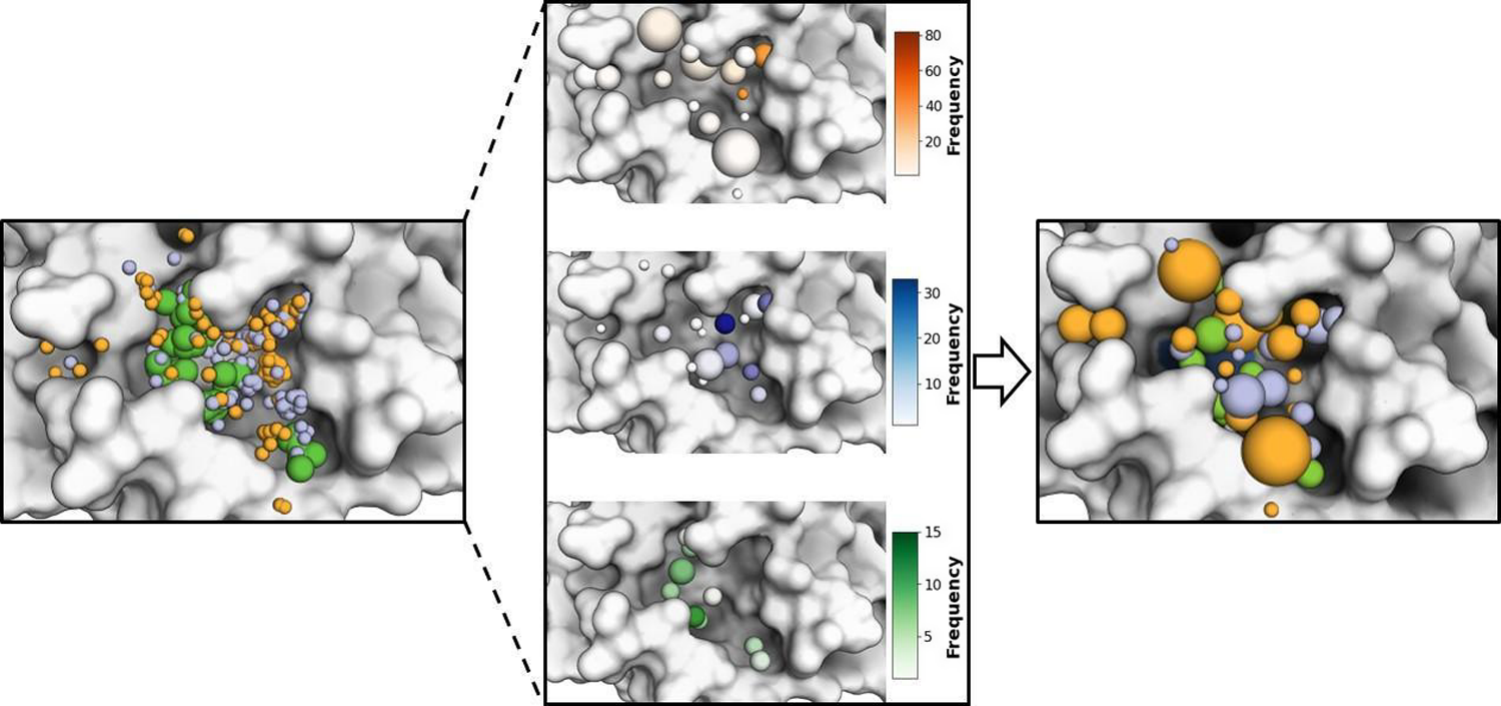
Consensus pharmacophore of the M^pro^catalytic site. The consensus pharmacophore generated includes 19 consensus descriptors for hydrogen bond acceptors (orange), 20 for hydrogen bond donors (blue), and 11 hydrophobic interactions (green). The generated model considers the cluster size (frequency), depicted as color intensity in the center panel, as well as the dispersion in the positions of the members of the cluster (radius), depicted as sphere size in the right panel.

The consensus points were combined to form a consensus model, which summarizes a large amount of structural and chemical information and represents all of the interaction types within the M^pro^ catalytic cavity (Figure 3). The final consensus pharmacophore had high complexity (19 descriptors for hydrogen bond acceptors, 20 for hydrogen bond donors, and 9 for hydrophobic interactions). By encompassing a large portion of the cavity, the model overcomes the limitations of individual pharmacophores, which may only capture a subset of the interaction potential. The coordinates of the consensus pharmacophore model are included in the Supporting Information (Table S2B).

The creation of a pharmacophore involves a series of steps that combine experimental data and computational techniques.^73^ A consensus pharmacophore is a model that combines the strengths of several individual pharmacophores to provide a more accurate and reliable representation of multiple molecules that bind to a common target. It is composed of geometric elements that represent molecular properties such as hydrophobic regions, hydrogen bond donors or acceptors, aromatic rings, and positive or negative charges. As a result, it can be used to guide the development of new antitarget compounds with comparable or improved activity. However, developing a consensus pharmacophore presents challenges, particularly in balancing the inclusion of common features with the risk of overlooking unique or context-specific interactions that may be critical for binding or specificity. The model presented in this study addresses this limitation by expanding the targeted protein area, thereby increasing the structural diversity of potential hits. This approach not only improves the utility of the model in identifying new inhibitors but also makes it a versatile tool that can be integrated with other computational methods (e.g., molecular docking or molecular dynamics) to refine the search for potent M^pro^ inhibitors and generate pharmacophores in drug discovery.

### 3.2 Consensus Pharmacophore Validation

**Figure 4:**
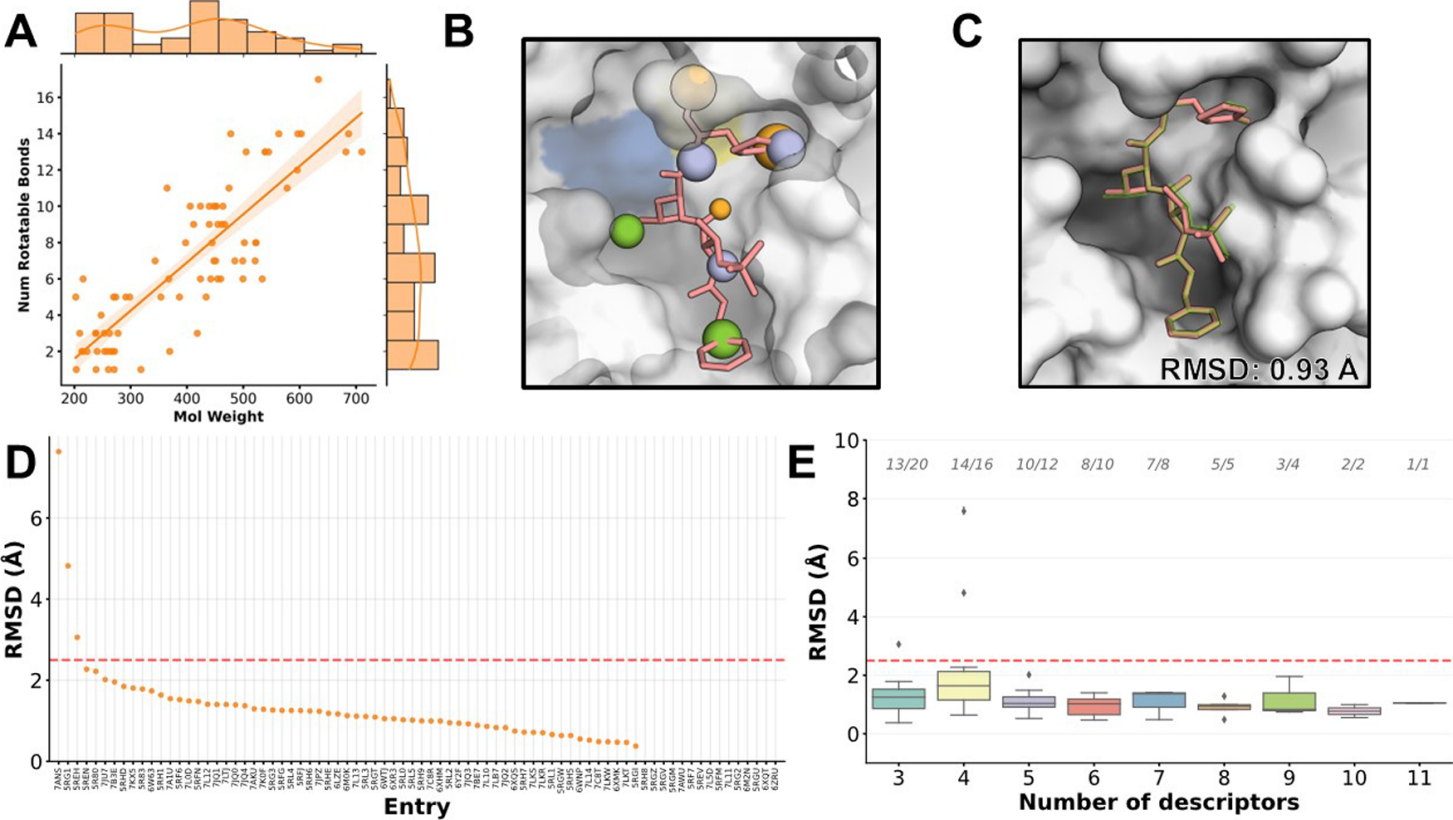
Validation of the consensus pharmacophore. (A) Number of rotatable bonds as a function of molecular weight for the 78 ligands included in the validation set. (B) Example of the pharmacophore-selected pose for one of the ligands from the validation set with the matching consensus descriptors from our model. Hydrophobic interactors, hydrogen bond acceptors, and hydrogen bond donors are colored green, orange, and purple, respectively. (C) Comparison of crystal (PDB ID: 7JQ3; green) and pharmacophore-driven (red) poses for the ligand shown in part B. (D) RMSD of all ligands within the validation set. (E) RMSD of poses predicted by the pharmacophore vs crystal poses, in subgroups of ligands with different numbers of descriptors.

During the validation phase of our study, we rigorously tested the consensus pharmacophore model against a curated library of conformers of 78 cocrystallized ligands drawn from the reference data set. These ligands represented molecules with a variety of molecular weights and rotatable bonds. As expected,^74^ we discovered a direct correlation between the number of rotatable bonds and molecular weight in our validation set (Figure 4A). This relationship emphasizes the complexity and diversity of the molecules evaluated, providing a robust test of the predictive capabilities of consensus pharmacophore. The pharmacophore-matching conformers were retrieved (Figure 4B) and their RMSD against the corresponding crystallographic poses was calculated (Figure 4C). Remarkably, the consensus pharmacophore model accurately recreated the crystallographic binding pose—with an RMSD < 2.5 Å—for 77% of the compounds in the validation set (Figure 4D). Whereas another 3.8% (three compounds) reproduced the crystallographic pose with RMSD > 2.5 Å, and the rest could not be retrieved. This high success rate was achieved regardless of the initial number of pharmacophoric descriptors identified for each ligand, demonstrating the robustness of the model (Figure 4E). The performance of *in silico* methods for predicting the correct poses depends on various factors, including the nature of the binding pocket (e.g., solvent-exposed or shallow pockets are challenging to identify) and the characteristics of the ligands (flexible ligands, such as peptides and macrocycles, are more difficult to model because of the large degrees of freedom). Moreover, the efficacy of these methods can be affected by the inclusion or omission of specific pharmacophore features and the application of constraints (e.g., covalent docking). In comparison, molecular docking, particularly when targeting M^pro^, has shown limited success in accurately replicating crystallized conformations, with success rates rarely exceeding 26%.^75,76^ This comparison highlights the superior performance of our consensus pharmacophore model in predicting ligand poses, confirming its potential utility in the discovery and optimization of novel inhibitors for SARS-CoV-2 M^pro^.

The consensus model generated included structural diversity from multiple ligands that covered all subpockets of the M^pro^ catalytic cavity. The complexity of our model, while making it highly comprehensive, required the generation of nine distinct submodels, each with seven to eight consensus pharmacophoric descriptors (Table S3A and Figure S4). This range is considered optimal for virtual screening applications, as it strikes a balance between specificity and generality.^77^ Although these submodels were created manually and based on extensive experience in pharmacophore development,^27,78^ they constitute a significant limitation in the current approach. However, we foresee the potential of machine learning or artificial intelligence^79^ to refine pharmacophore modeling, making the process more efficient and less reliant on manual intervention.

**Figure 5:**
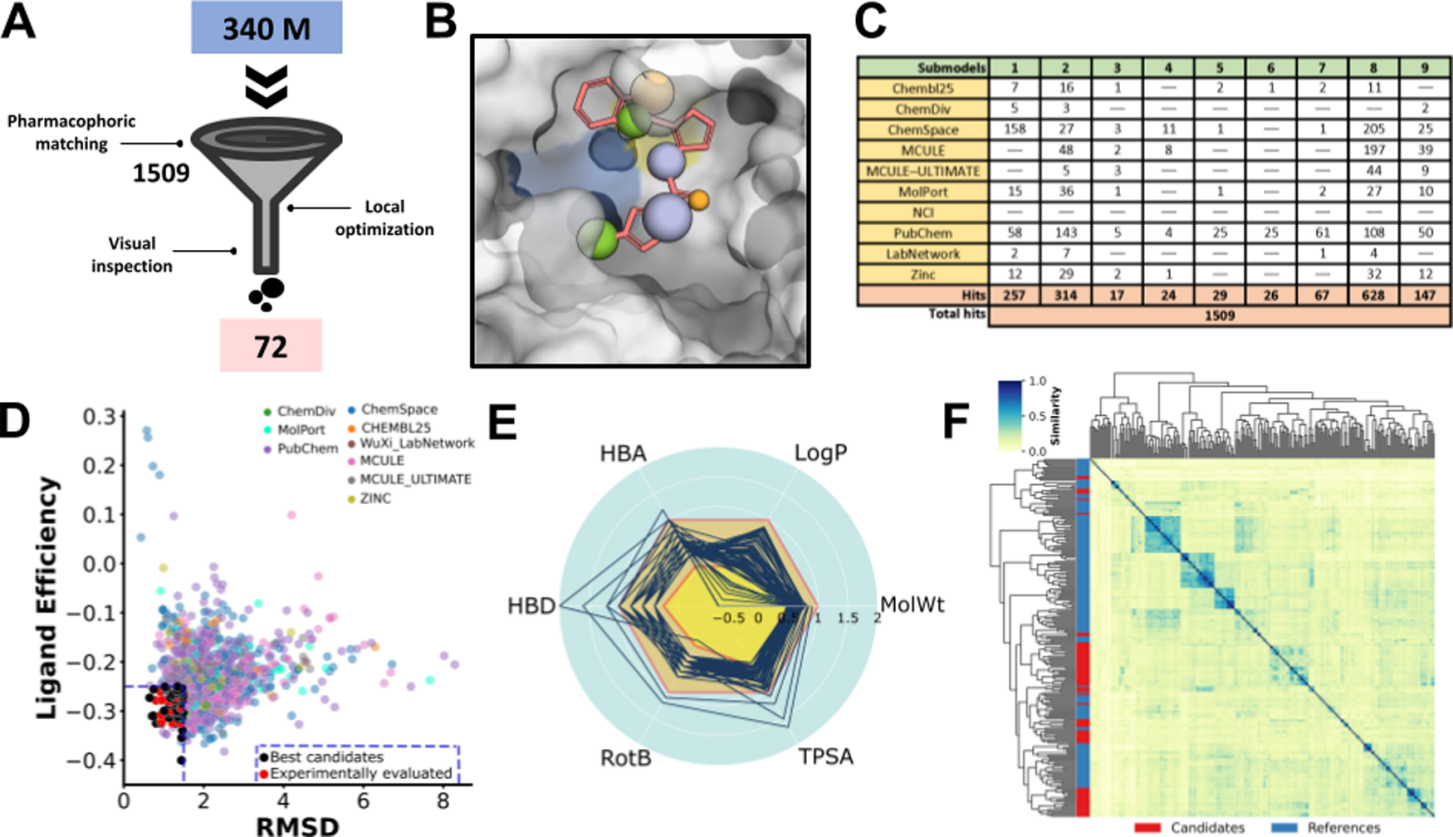
Identification of potential M^pro^inhibitors. (A) General scheme for candidate selection. (B) Pharmacophore-matching of the candidate compound, PubChem-136601252. (C) Number of hits obtained from different chemical libraries using nine pharmacophore submodels. (D) LE vs RMSD of poses before and after local optimization for the hit compounds. We selected compounds (black dots) with LE < −0.25, RMSD < 1.5 Å, and at least 6 descriptors (dotted lines) that passed visual inspection. From the candidates, 16 molecules were selected for experimental validation (red dots; E) Radar plot of physicochemical properties for the selected potential M^pro^ inhibitors. (F) Similarity matrix of the selected compounds. The compounds in the reference set were included for comparison.

Our virtual screening process, which relied on the consensus pharmacophore model, efficiently processed 343,353,042 compounds from publicly available chemical libraries (Figure 5A,B), demonstrating the model’s capability to manage ultralarge chemical spaces with minimal computational demands. This process yielded 1509 pharmacophore hits (Figure 5C), which were further filtered based on ligand efficiency (LE) and RMSD (pharmacophore-driven pose vs obtained pose after local optimization) criteria, emphasizing the selection of compounds that efficiently use their atoms in binding to the target (Figure 5D).^80^ Subsequent filtering excluded compounds with known M^pro^ activity or those with steric clashes between the ligands and protein surface, resulting in 72 candidate compounds with drug-like physicochemical properties (Table S3B, and Figure 5E). Notably, these candidates are structurally diverse, from both the reference set and among themselves (Figure 5F). This diversity is important because it suggests that our model can identify and discover novel scaffolds for M^pro^ inhibition, as opposed to previously discovered inhibitors that frequently share common structural motifs such as flavonoids, hydroxyethylamine analogs, and naphthoquinones derivatives.^81−83^ Besides, the obtained chemical diversity pinpoints the innovative potential of the consensus pharmacophore model in broadening the scope of M^pro^ inhibitor discovery beyond conventional chemotypes.

### 3.3 Experimental Validation

**Figure 6:**
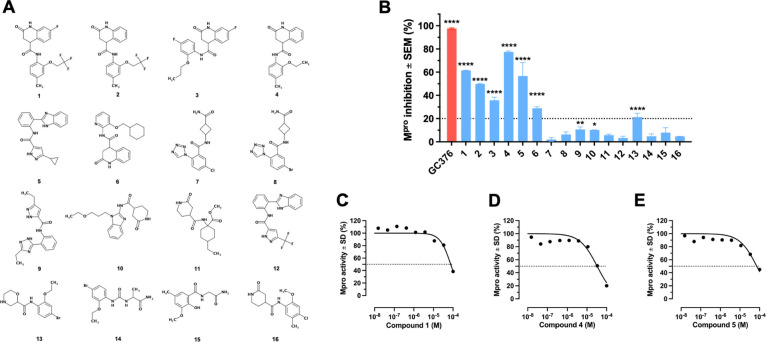
Experimental evaluation of computational hits. (A) Structure of compounds 1–16. (B) Evaluation of the effect of GC376 (positive control) and compounds 1–16 on M^pro^ activity. Error bars shown represent the standard error of the mean (SEM) from two independent experiments. *P < 0.05; **P < 0.01; *** *P* < 0.001; ****P < 0.0001 (Dunnett’s tests vs vehicle control). (C–E) Dose–response curves for compounds 1 (C), 4 (D), and 5 (E) determined by a FRET-based cleavage assay.

To identify effective M^pro^ inhibitors, we purchased 16 compounds (Figure 6A) identified by using our consensus pharmacophore model. Remarkably, seven out of the 16 evaluated compounds exhibited inhibition of M^pro^ catalytic activity >20% (Figure 6B), demonstrating the applicability of our pharmacophore model. We further characterized the activities of the three most active compounds. Compounds 1, 4, and 5 matched submodels that fit the S1, S1’, and S2 subpockets (Figure S5A–C) and inhibited M^pro^ activity with IC_50_ values of 86.6, 32.6, and 70.5 μM, respectively (Figure 6C–E). The three compounds have inhibitory potencies that are comparable, if not superior, to those of other M^pro^ inhibitors discovered using virtual screening methods. For instance, a natural compound, W7, was previously identified with an IC_50_ of 75 μM through a pharmacophore-based approach.^84^ Similarly, molecular docking and MD simulations helped to identify inhibitors with IC_50_ values ranging from 6.74 to 1370 μM.^85^ These comparisons validate the efficacy of our screening strategy and underscore the strength of our consensus pharmacophore model in identifying viable M^pro^ inhibitors.

### 3.4 Characterization of M^pro^ Binding Modes

**Figure 7:**
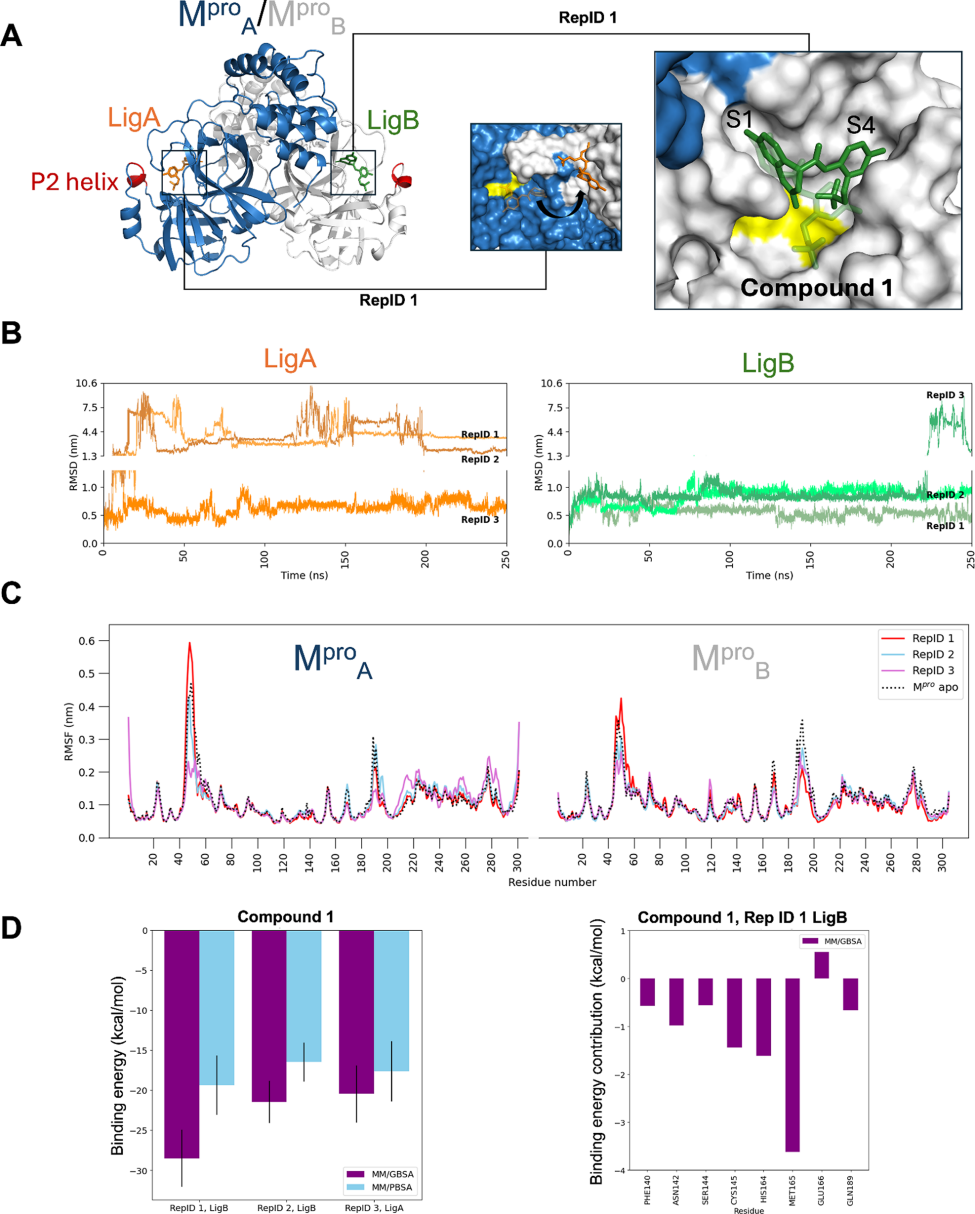
Molecular dynamics simulations of the M^pro^/compound 1 complex. (A) Analyzed complex comprised two M^pro^ protomers: M^pro^_A_ as blue and M^pro^_B_ as white. Each catalytic cavity had one ligand (LigA and LigB, shown as stick models in orange and green, respectively). The insets display the initial conformation (transparent sticks) and a representative conformer from the MD simulation (solid sticks). The catalytic dyad is depicted in yellow, and the P2 helix is highlighted in red for both monomers. (B) RMSD graphs for compound 1 (LigA and LigB) in three different replicates of the MD simulation. (C) RMSF graphs for alpha-carbons of M^pro^ protomers A and B with ligands in three different replicates of MD simulation. The M^pro^ dimer without a ligand (apo) is presented for comparison. (D) Binding energy calculated for the ligands that remained bound using the molecular mechanics generalized Born surface area (MM/GBSA) and Poisson–Boltzmann surface area (MM/PBSA) methods. (E) Contribution by residue to binding energy for LigB in RepID 1 by MM/GBSA.

We performed MD simulations on the M^pro^ dimer complexed with compounds 1, 4, or 5. Each system contained two identical ligands (LigA and LigB), each one bound to one of the M^pro^ protomers (M^pro^_A_ or M^pro^_B_). In general, we observed that only one of the ligands maintained its position within the M^pro^ catalytic cavity across three MD simulation replicates (Figures 7A, S6A and S7A), indicating a partial dissociation phenomenon. The average RMSD for the ligands that remained bound to the catalytic site varied between 5.7 and 10.9 Å. Despite this variation, they showed stable standard deviations (±1.1 to 3.3 Å) suggesting that the ligands, once settled into new conformations, maintain those positions reliably (Figures 7B, S6B and S7B). On the other hand, the backbone RMSD of M^pro^ showed minimal structural changes, indicating that the protein is stable in the presence of these ligands (Figure S8A–C).

The Root-mean-square fluctuation (RMSF) of the M^pro^ dimeric structure showed a notable divergence in the behavior of the P2 helix across protomers. The protomer with ligand leaving the catalytic cavity exhibited high RMSF in the P2 helix, indicating that it displayed divergent conformations. In contrast, the monomer retaining the ligand had reduced RMSF in the residues forming the P2 helix, indicating that the stable interaction with the ligand restricted its conformational dynamics (Figures 7C, S6C and S7C). This differential behavior suggests a potential long-distance mechanism in which the binding of a ligand to one protomer may influence the conformation of the binding site in the other, potentially affecting the capability of M^pro^ to allocate two ligands simultaneously. This observation is consistent with the role of distortion and malleability of the catalytic site described for the P2 helix.^86^

The calculated binding energy for the stably bound ligands showed average values ranging from −30.59 to −8.58 kcal/mol (Figures 7D, S6D and S7D), supporting the efficacy of these compounds as M^pro^ inhibitors. Examination of the energy contributions per residue found a key role of Met165 and the catalytic residue Cys145 in maintaining the binding of compounds 1 and 4 (Figures 7E and S6E). His41, the second catalytic residue, mediated a favorable interaction with compounds 4 and 5 (Figures S6E and S7E). Finally, Met49, part of the P2 helix, and Gln189 established contacts with compound 5 (Figure S7E). Interactions of M^pro^ inhibitors with the catalytic residues are desirable given the direct effect elicited on enzymatic activity.^87,88^ Similarly, targeting residues on S1 favors stable binding of M^pro^ inhibitors because of the conformational stability of the subcavity.^86^

The residues mediating the binding of compounds 1, 4, and 5 are highly conserved among circulating SARS-CoV-2 variants, displaying substitution frequencies <0.028%.^89^ However, mutations at Met49 and Met165 reduce the inhibitory activity of ensitrelvir and nilmatrevir, respectively,^90^ and could also impact the activity of the new M^pro^ inhibitors described here. In contrast, Glu166 did not favor ligand binding; thus, we hypothesize that substitutions on such residue, which impair ensitrelvir and nilmatrevir efficacy,^90^ would have a limited effect on the activity of our compounds.

The observed dissociation of ligands from the M^pro^ dimer, the differences between initial and stable conformations of ligands that remained bound, and the complex role of protein dynamics in ligand stability indicate that the inhibitors reported here require additional optimization.

## 4 Conclusions

We developed and validated a consensus pharmacophore from 152 bioactive conformers cocrystallized with SARS-CoV-2 M^pro^. Our new model reduces the number of pharmacophoric descriptors by clustering them by type of interaction, spatial position, and frequency of appearance. The identification of 72 candidate compounds through this process emphasizes the practical utility of our model in screening vast chemical libraries to pinpoint molecules with significant inhibitory potential against M^pro^. Experimental evaluation of a subset of candidate compounds identified multiple M^pro^ inhibitors, the most active with IC_50_ values from 32.6 to 86.6 μM. Furthermore, our automated consensus pharmacophore generation technique is freely available, democratizing access to this methodology and allowing academics to use it on their own data sets. This strategy not only makes it easier to find new M^pro^ inhibitors but also is flexible and adaptable to be used to other pharmacological targets that pose comparable difficulties, including numerous ligand-target complexes or intricate catalytic cavities.

## Data Availability

Data supporting the reported results is included as Supporting Information. The method developed for consensus pharmacophore generation is freely available at https://github.com/AngelRuizMoreno/ConcensusPharmacophore.

## Supporting Information Available

Number of M^pro^ structures available in PDB over time, identification of consensus pharmacophoric points for hydrogen bond donors, identification of consensus pharmacophoric points for hydrogen bond acceptors, pharmacophoric submodels generated from the consensus pharmacophore, pharmacophore matching of the candidate compounds 1, 4, and 5, analysis of MD simulations for complex M^pro^-compound 4, analysis of MD simulations for complex M^pro^-compound 5, and RMSD of M^pro^ alpha-carbons from MD simulations with compounds 1, 4, and 5 (PDF)
Spreadsheets are provided containing information on the M^pro^ crystal structures and ligands in the reference set (XLSX)
Pharmacophoric descriptors obtained from the reference set and the descriptors and coordinates of the consensus pharmacophore (XLSX)
Submodels employed for virtual screening and the potential M^pro^ inhibitors identified (XLSX)

## References

[ref1] Huang C.; Wang Y.; Li X.; Ren L.; Zhao J.; Hu Y.; Zhang L.; Fan G.; Xu J.; Gu X.; Cheng Z.; Yu T.; Xia J.; Wei Y.; Wu W.; Xie X.; Yin W.; Li H.; Liu M.; Xiao Y.; Gao H.; Guo L.; Xie J.; Wang G.; Jiang R.; Gao Z.; Jin Q.; Wang J.; Cao B. Clinical Features of Patients Infected with 2019 Novel Coronavirus in Wuhan. China. The Lancet 2020, 395, 497–506. 10.1016/S0140-6736(20)30183-5. PMC7159299 31986264

[ref2] Tahir Ul Qamar M.; Alqahtani S. M.; Alamri M. A.; Chen L.-L Structural Basis of SARS-CoV-2 3CLpro and Anti-COVID-19 Drug Discovery from Medicinal Plants. J. Pharm. Anal. 2020, 10, 313–319. 10.1016/j.jpha.2020.03.009. 32296570 PMC7156227

[ref3] Adil M. T.; Rahman R.; Whitelaw D.; Jain V.; Al-Taan O.; Rashid F.; Munasinghe A.; Jambulingam P. SARS-CoV-2 and the Pandemic of COVID-19. Postgrad. Med. J. 2021, 97, 110–116. 10.1136/postgradmedj-2020-138386. 32788312 PMC10016996

[ref4] Zhou P.; Yang X.-L.; Wang X.-G.; Hu B.; Zhang L.; Zhang W.; Si H.-R.; Zhu Y.; Li B.; Huang C.-L.; Chen H.-D.; Chen J.; Luo Y.; Guo H.; Jiang R.-D.; Liu M.-Q.; Chen Y.; Shen X.-R.; Wang X.; Zheng X.-S.; Zhao K.; Chen Q.-J.; Deng F.; Liu L.-L.; Yan B.; Zhan F.-X.; Wang Y.-Y.; Xiao G.-F.; Shi Z.-L. A Pneumonia Outbreak Associated with a New Coronavirus of Probable Bat Origin. Nature 2020, 579, 270–273. 10.1038/s41586-020-2012-7. 32015507 PMC7095418

[ref5] Wu F.; Zhao S.; Yu B.; Chen Y.-M.; Wang W.; Song Z.-G.; Hu Y.; Tao Z.-W.; Tian J.-H.; Pei Y.-Y.; Yuan M.-L.; Zhang Y.-L.; Dai F.-H.; Liu Y.; Wang Q.-M.; Zheng J.-J.; Xu L.; Holmes E. C.; Zhang Y.-Z. A New Coronavirus Associated with Human Respiratory Disease in China. Nature 2020, 579 (7798), 265–269. 10.1038/s41586-020-2008-3. 32015508 PMC7094943

[ref6] Zhu N.; Zhang D.; Wang W.; Li X.; Yang B.; Song J.; Zhao X.; Huang B.; Shi W.; Lu R.; Niu P.; Zhan F.; Ma X.; Wang D.; Xu W.; Wu G.; Gao G. F.; Tan W. A Novel Coronavirus from Patients with Pneumonia in China, 2019. N. Engl. J. Med. 2020, 382, 727–733. 10.1056/NEJMoa2001017. 31978945 PMC7092803

[ref7] Perlman S.; Netland J. Coronaviruses Post-SARS: Update on Replication and Pathogenesis. Nat. Rev. Microbiol. 2009, 7 (6), 439–450. 10.1038/nrmicro2147. 19430490 PMC2830095

[ref8] Fehr A. R.; Perlman S. Coronaviruses: An Overview of Their Replication and Pathogenesis. In Coronaviruses: Methods and Protocols; Maier H. J.; Bickerton E.; Britton P., Eds.; Methods in Molecular Biology; Springer: New York, NY, 2015; 1–23. 10.1007/978-1-4939-2438-7_1 PMC4369385 25720466

[ref9] Anand K.; Ziebuhr J.; Wadhwani P.; Mesters J.; Hilgenfeld R. Coronavirus Main Proteinase (3CLpro) Structure: Basis for Design of Anti-SARS Drugs. Science 2003, 300, 1763–1767. 10.1126/science.1085658. 12746549

[ref10] Mielech A. M.; Chen Y.; Mesecar A. D.; Baker S. C. Nidovirus Papain-like Proteases: Multifunctional Enzymes with Protease Deubiquitinating and deISGylating Activities. Virus Res. 2014, 194, 184–190. 10.1016/j.virusres.2014.01.025. 24512893 PMC4125544

[ref11] Melo-Filho C. C.; Bobrowski T.; Martin H.-J.; Sessions Z.; Popov K. I.; Moorman N. J.; Baric R. S.; Muratov E. N.; Tropsha A. Conserved Coronavirus Proteins as Targets of Broad-Spectrum Antivirals. Antiviral Res. 2022, 204, 105360 10.1016/j.antiviral.2022.105360. 35691424 PMC9183392

[ref12] Rut W.; Groborz K.; Zhang L.; Sun X.; Zmudzinski M.; Pawlik B.; Wang X.; Jochmans D.; Neyts J.; Młynarski W.; Hilgenfeld R.; Drag M. SARS-CoV-2 Mpro Inhibitors and Activity-Based Probes for Patient-Sample Imaging. Nat. Chem. Biol. 2021, 17, 222–228. 10.1038/s41589-020-00689-z. 33093684

[ref13] Jin Z.; Du X.; Xu Y.; Deng Y.; Liu M.; Zhao Y.; Zhang B.; Li X.; Zhang L.; Peng C.; Duan Y.; Yu J.; Wang L.; Yang K.; Liu F.; Jiang R.; Yang X.; You T.; Liu X.; Yang X.; Bai F.; Liu H.; Liu X.; Guddat L. W.; Xu W.; Xiao G.; Qin C.; Shi Z.; Jiang H.; Rao Z.; Yang H. Structure of Mpro from SARS-CoV-2 and Discovery of Its Inhibitors. Nature 2020, 582, 289–293. 10.1038/s41586-020-2223-y. 32272481

[ref14] Lee J.; Worrall L. J.; Vuckovic M.; Rosell F. I.; Gentile F.; Ton A.-T.; Caveney N. A.; Ban F.; Cherkasov A.; Paetzel M.; Strynadka N. C. J. Crystallographic Structure of Wild-Type SARS-CoV-2 Main Protease Acyl-Enzyme Intermediate with Physiological C-Terminal Autoprocessing Site. Nat. Commun. 2020, 11, 5877 10.1038/s41467-020-19662-4. 33208735 PMC7674412

[ref15] Singh E.; Khan R. J.; Jha R. K.; Amera G. M.; Jain M.; Singh R. P.; Muthukumaran J.; Singh A. K. A Comprehensive Review on Promising Anti-Viral Therapeutic Candidates Identified against Main Protease from SARS-CoV-2 through Various Computational Methods. J. Genet. Eng. Biotechnol. 2020, 18, 69 10.1186/s43141-020-00085-z. 33141358 PMC7607901

[ref16] Lee J.; Kenward C.; Worrall L. J.; Vuckovic M.; Gentile F.; Ton A.-T.; Ng M.; Cherkasov A.; Strynadka N. C. J.; Paetzel M. X-Ray Crystallographic Characterization of the SARS-CoV-2 Main Protease Polyprotein Cleavage Sites Essential for Viral Processing and Maturation. Nat. Commun. 2022, 13, 5196 10.1038/s41467-022-32854-4. 36057636 PMC9440467

[ref17] Roe M. K.; Junod N. A.; Young A. R.; Beachboard D. C.; Stobart C. C. Targeting Novel Structural and Functional Features of Coronavirus Protease Nsp5 (3CLpro, Mpro) in the Age of COVID-19. J. Gen. Virol. 2021, 102, 001558 10.1099/jgv.0.001558. 33507143 PMC8515871

[ref18] Gliptin Repurposing for COVID-19 | Biological and Medicinal Chemistry | ChemRxiv | Cambridge Open Engage. https://chemrxiv.org/engage/chemrxiv/article-details/60c749dd469df4d40af43c4d (accessed 2023–08–23).

[ref19] Hosseini M.; Chen W.; Xiao D.; Wang C. Computational Molecular Docking and Virtual Screening Revealed Promising SARS-CoV-2 Drugs. Precis. Clin. Med. 2021, 4, 1–16. 10.1093/pcmedi/pbab001. 33842834 PMC7928605

[ref20] Jiménez-Alberto A.; Ribas-Aparicio R. M.; Aparicio-Ozores G.; Castelán-Vega J. A. Virtual Screening of Approved Drugs as Potential SARS-CoV-2 Main Protease Inhibitors. Comput. Biol. Chem. 2020, 88, 107325 10.1016/j.compbiolchem.2020.107325. 32623357 PMC7316061

[ref21] Elseginy S. A. Virtual Screening and Structure-Based 3D Pharmacophore Approach to Identify Small-Molecule Inhibitors of SARS-CoV-2 Mpro. J. Biomol. Struct. Dyn. 2022, 40 (24), 13658–13674. 10.1080/07391102.2021.1993341. 34676801

[ref22] Gahlawat A.; Kumar N.; Kumar R.; Sandhu H.; Singh I. P.; Singh S.; Sjöstedt A.; Garg P. Structure-Based Virtual Screening to Discover Potential Lead Molecules for the SARS-CoV-2 Main Protease. J. Chem. Inf. Model. 2020, 60, 5781–5793. 10.1021/acs.jcim.0c00546. 32687345

[ref23] Unoh Y.; Uehara S.; Nakahara K.; Nobori H.; Yamatsu Y.; Yamamoto S.; Maruyama Y.; Taoda Y.; Kasamatsu K.; Suto T.; Kouki K.; Nakahashi A.; Kawashima S.; Sanaki T.; Toba S.; Uemura K.; Mizutare T.; Ando S.; Sasaki M.; Orba Y.; Sawa H.; Sato A.; Sato T.; Kato T.; Tachibana Y. Discovery of S-217622, a Noncovalent Oral SARS-CoV-2 3CL Protease Inhibitor Clinical Candidate for Treating COVID-19. J. Med. Chem. 2022, 65, 6499–6512. 10.1021/acs.jmedchem.2c00117. 35352927 PMC8982737

[ref24] Dawood A. A. The Efficacy of Paxlovid against COVID-19 Is the Result of the Tight Molecular Docking between Mpro and Antiviral Drugs (Nirmatrelvir and Ritonavir). Adv. Med. Sci. 2023, 68, 1–9. 10.1016/j.advms.2022.10.001. 36368287 PMC9626444

[ref25] Chatterjee S.; Bhattacharya M.; Dhama K.; Lee S.-S.; Chakraborty C. Resistance to Nirmatrelvir Due to Mutations in the Mpro in the Subvariants of SARS-CoV-2 Omicron: Another Concern?. Mol. Ther. - Nucleic Acids 2023, 32, 263–266. 10.1016/j.omtn.2023.03.013. 37041859 PMC10078092

[ref26] Jin X.; Zhang M.; Fu B.; Li M.; Yang J.; Zhang Z.; Li C.; Zhang H.; Wu H.; Xue W.; Liu Y. Structure-Based Discovery of the SARS-CoV-2 Main Protease Noncovalent Inhibitors from Traditional Chinese Medicine. J. Chem. Inf. Model. 2024, 64, 1319 10.1021/acs.jcim.3c01327. 38346323

[ref27] Sadremomtaz A.; Al-Dahmani Z. M.; Ruiz-Moreno A. J.; Monti A.; Wang C.; Azad T.; Bell J. C.; Doti N.; Velasco-Velázquez M. A.; de Jong D.; de Jonge J.; Smit J.; Dömling A.; van Goor H.; Groves M. R. Synthetic Peptides That Antagonize the Angiotensin-Converting Enzyme-2 (ACE-2) Interaction with SARS-CoV-2 Receptor Binding Spike Protein. J. Med. Chem. 2022, 65, 2836–2847. 10.1021/acs.jmedchem.1c00477. 34328726 PMC8353989

[ref28] Nawrot-Hadzik I.; Zmudzinski M.; Matkowski A.; Preissner R.; Kęsik-Brodacka M.; Hadzik J.; Drag M.; Abel R. Reynoutria Rhizomes as a Natural Source of SARS-CoV-2 Mpro Inhibitors–Molecular Docking and In Vitro Study. Pharmaceuticals 2021, 14, 742 10.3390/ph14080742. 34451839 PMC8399519

[ref29] Zhu Y.; Xie D.-Y. Docking Characterization and in Vitro Inhibitory Activity of Flavan-3-Ols and Dimeric Proanthocyanidins Against the Main Protease Activity of SARS-Cov-2. Front. Plant Sci. 2020, 11, 601316 10.3389/fpls.2020.601316. 33329667 PMC7733993

[ref30] Abo Elmaaty A.; Eldehna W. M.; Khattab M.; Kutkat O.; Alnajjar R.; El-Taweel A. N.; Al-Rashood S. T.; Abourehab M. A. S.; Binjubair F. A.; Saleh M. A.; Belal A.; Al-Karmalawy A. A. Anticoagulants as Potential SARS-CoV-2 Mpro Inhibitors for COVID-19 Patients: In Vitro, Molecular Docking, Molecular Dynamics, DFT, and SAR Studies. Int. J. Mol. Sci. 2022, 23, 12235 10.3390/ijms232012235. 36293094 PMC9603561

[ref31] Aljuhani A.; Ahmed H. E. A.; Ihmaid S. K.; Omar A. M.; Althagfan S. S.; Alahmadi Y. M.; Ahmad I.; Patel H.; Ahmed S.; Almikhlafi M. A.; El Agrody A. M.; Zayed M. F.; Abdulrahman Turkistani S.; Abulkhair S. H.; Almaghrabi M.; Salama S. A.; Al Karmalawy A. A.; Abulkhair H. S. In Vitro and Computational Investigations of Novel Synthetic Carboxamide-Linked Pyridopyrrolopyrimidines with Potent Activity as SARS-CoV-2-M Pro Inhibitors. RSC Adv. 2022, 12, 26895–26907. 10.1039/D2RA04015H. 36320844 PMC9494209

[ref32] Alhadrami H. A.; Burgio G.; Thissera B.; Orfali R.; Jiffri S. E.; Yaseen M.; Sayed A. M.; Rateb M. E. Neoechinulin A as a Promising SARS-CoV-2 Mpro Inhibitor: In Vitro and In Silico Study Showing the Ability of Simulations in Discerning Active from Inactive Enzyme Inhibitors. Mar. Drugs 2022, 20, 163 10.3390/md20030163. 35323462 PMC8955780

[ref33] Alves V. M.; Bobrowski T.; Melo-Filho C. C.; Korn D.; Auerbach S.; Schmitt C.; Muratov E. N.; Tropsha A. QSAR Modeling of SARS-CoV Mpro Inhibitors Identifies Sufugolix, Cenicriviroc, Proglumetacin, and Other Drugs as Candidates for Repurposing against SARS-CoV-2. Mol. Inform. 2021, 40, 2000113 10.1002/minf.202000113. 33405340

[ref34] Wang L.; Bao B.-B.; Song G.-Q.; Chen C.; Zhang X.-M.; Lu W.; Wang Z.; Cai Y.; Li S.; Fu S.; Song F.-H.; Yang H.; Wang J.-G. Discovery of Unsymmetrical Aromatic Disulfides as Novel Inhibitors of SARS-CoV Main Protease: Chemical Synthesis, Biological Evaluation, Molecular Docking and 3D-QSAR Study. Eur. J. Med. Chem. 2017, 137, 450–461. 10.1016/j.ejmech.2017.05.045. 28624700 PMC7115414

[ref35] Guevara-Pulido J.; Jiménez R. A.; Morantes S. J.; Jaramillo D. N.; Acosta-Guzmán P. Design, Synthesis, and Development of 4-[(7-Chloroquinoline-4-Yl)Amino]Phenol as a Potential SARS-CoV-2 Mpro Inhibitor. ChemistrySelect 2022, 7, e202200125 10.1002/slct.202200125. 35601684 PMC9111044

[ref36] Juárez-Mercado K. E.; Gómez-Hernández M. A.; Salinas-Trujano J.; Córdova-Bahena L.; Espitia C.; Pérez-Tapia S. M.; Medina-Franco J. L.; Velasco-Velázquez M. A. Identification of SARS-CoV-2 Main Protease Inhibitors Using Chemical Similarity Analysis Combined with Machine Learning. Pharmaceuticals 2024, 17, 240 10.3390/ph17020240. 38399455 PMC10892746

[ref37] Elagawany M.; Elmaaty A. A.; Mostafa A.; Abo Shama N. M.; Santali E. Y.; Elgendy B.; Al-Karmalawy A. A. Ligand-Based Design, Synthesis, Computational Insights, and in Vitro Studies of Novel N-(5-Nitrothiazol-2-Yl)-Carboxamido Derivatives as Potent Inhibitors of SARS-CoV-2 Main Protease. J. Enzyme Inhib. Med. Chem. 2022, 37, 2112–2132. 10.1080/14756366.2022.2105322. 35912578 PMC9344964

[ref38] Mercorelli B.; Desantis J.; Celegato M.; Bazzacco A.; Siragusa L.; Benedetti P.; Eleuteri M.; Croci F.; Cruciani G.; Goracci L.; Loregian A. Discovery of Novel SARS-CoV-2 Inhibitors Targeting the Main Protease Mpro by Virtual Screenings and Hit Optimization. Antiviral Res. 2022, 204, 105350 10.1016/j.antiviral.2022.105350. 35688349 PMC9172283

[ref39] Li Z.; Li X.; Huang Y.-Y.; Wu Y.; Liu R.; Zhou L.; Lin Y.; Wu D.; Zhang L.; Liu H.; Xu X.; Yu K.; Zhang Y.; Cui J.; Zhan C.-G.; Wang X.; Luo H.-B. Identify Potent SARS-CoV-2 Main Protease Inhibitors via Accelerated Free Energy Perturbation-Based Virtual Screening of Existing Drugs. Proc. Natl. Acad. Sci. U. S. A. 2020, 117 (44), 27381–27387. 10.1073/pnas.2010470117. 33051297 PMC7959488

[ref40] Duong C. Q.; Nguyen P. T. V. Exploration of SARS-CoV-2 Mpro Noncovalent Natural Inhibitors Using Structure-Based Approaches. ACS Omega 2023, 8, 6679–6688. 10.1021/acsomega.2c07259. 36844600 PMC9947982

[ref41] Gossen J.; Albani S.; Hanke A.; Joseph B. P.; Bergh C.; Kuzikov M.; Costanzi E.; Manelfi C.; Storici P.; Gribbon P.; Beccari A. R.; Talarico C.; Spyrakis F.; Lindahl E.; Zaliani A.; Carloni P.; Wade R. C.; Musiani F.; Kokh D. B.; Rossetti G. A Blueprint for High Affinity SARS-CoV-2 Mpro Inhibitors from Activity-Based Compound Library Screening Guided by Analysis of Protein Dynamics. ACS Pharmacol. Transl. Sci. 2021, 4, 1079–1095. 10.1021/acsptsci.0c00215. 34136757 PMC8009102

[ref42] Alhadrami H. A.; Sayed A. M.; Al-Khatabi H.; Alhakamy N. A.; Rateb M. E. Scaffold Hopping of α-Rubromycin Enables Direct Access to FDA-Approved Cromoglicic Acid as a SARS-CoV-2 MPro Inhibitor. Pharmaceuticals 2021, 14, 541 10.3390/ph14060541. 34198933 PMC8229550

[ref43] Hayek-Orduz Y.; Vásquez A. F.; Villegas-Torres M. F.; Caicedo P. A.; Achenie L. E. K.; González Barrios A. F. Novel Covalent and Non-Covalent Complex-Based Pharmacophore Models of SARS-CoV-2 Main Protease (Mpro) Elucidated by Microsecond MD Simulations. Sci. Rep. 2022, 12, 14030 10.1038/s41598-022-17204-0. 35982147 PMC9386674

[ref44] Murumkar P. R.; Sharma M. K.; Gupta P.; Patel N. M.; Yadav M. R. Selection of Suitable Protein Structure from Protein Data Bank: An Important Step in Structure-Based Drug Design Studies. Mini Rev. Med. Chem. 2023, 23, 246–264. 10.2174/1389557522666220512151454. 35549880

[ref45] UniProt: The Universal Protein Knowledgebase in 2021. Nucleic Acids Res. 2021, 49, D480–D489. 10.1093/nar/gkaa1100. 33237286 PMC7778908

[ref46] Sunseri J.; Koes D. R. Pharmit: Interactive Exploration of Chemical Space. Nucleic Acids Res. 2016, 44, W442–W448. 10.1093/nar/gkw287. 27095195 PMC4987880

[ref47] Schrödinger L.; DeLano W.. PyMOL, 2020. http://www.pymol.org/pymol.

[ref48] Whitmer J. C.; Cyvin S. J.; Cyvin B. N. Harmonie Force Fields and Bond Orders for Naphthalene, Anthracene, Biphenylene and Perylene with Mean Amplitudes for Perylene. Z. Für Naturforschung A 1978, 33, 45–54. 10.1515/zna-1978-0110.

[ref49] Gunbas G.; Hafezi N.; Sheppard W. L.; Olmstead M. M.; Stoyanova I. V.; Tham F. S.; Meyer M. P.; Mascal M. Extreme Oxatriquinanes and a Record C–O Bond Length. Nat. Chem. 2012, 4, 1018–1023. 10.1038/nchem.1502. 23174982

[ref50] Zhang H.; Jiang X.; Wu W.; Mo Y. Electron Conjugation versus π–π Repulsion in Substituted Benzenes: Why the Carbon–Nitrogen Bond in Nitrobenzene Is Longer than in Aniline. Phys. Chem. Chem. Phys. 2016, 18, 11821–11828. 10.1039/C6CP00471G. 26852720

[ref51] Gaulton A.; Hersey A.; Nowotka M.; Bento A. P.; Chambers J.; Mendez D.; Mutowo P.; Atkinson F.; Bellis L. J.; Cibrián-Uhalte E.; Davies M.; Dedman N.; Karlsson A.; Magariños M. P.; Overington J. P.; Papadatos G.; Smit I.; Leach A. R. The ChEMBL Database in 2017. Nucleic Acids Res. 2017, 45, D945–D954. 10.1093/nar/gkw1074. 27899562 PMC5210557

[ref52] CHEMDIV INC - Fully Integrated Target-To-Clinic Contract Research Organization (CRO). https://www.chemdiv.com/ (accessed 2023–08–24).

[ref53] Chemspace - the largest catalog of small molecules and biologics. https://chem-space.com/ (accessed 2023–08–24).

[ref54] Kiss R.; Sandor M.; Szalai F. A. A Public Web Service for Drug Discovery. J. Cheminformatics 2012, 4, P17 10.1186/1758-2946-4-S1-P17.

[ref55] Mcule - Ultimate Database Project. https://ultimate.mcule.com/ (accessed 2023–08–24).

[ref56] Compound Sourcing, Selling and Purchasing Platform. Molport. https://www.molport.com/shop/index (accessed 2023–08–24).

[ref57] Voigt J. H.; Bienfait B.; Wang S.; Nicklaus M. C. Comparison of the NCI Open Database with Seven Large Chemical Structural Databases. J. Chem. Inf. Comput. Sci. 2001, 41, 702–712. 10.1021/ci000150t. 11410049

[ref58] Kim S.; Thiessen P. A.; Bolton E. E.; Chen J.; Fu G.; Gindulyte A.; Han L.; He J.; He S.; Shoemaker B. A.; Wang J.; Yu B.; Zhang J.; Bryant S. H. PubChem Substance and Compound Databases. Nucleic Acids Res. 2016, 44, D1202–D1213. 10.1093/nar/gkv951. 26400175 PMC4702940

[ref59] LabNetwork. https://www.labnetwork.com/frontend-app/p/#!/ (accessed 2023–08–24).

[ref60] Irwin J. J.; Tang K. G.; Young J.; Dandarchuluun C.; Wong B. R.; Khurelbaatar M.; Moroz Y. S.; Mayfield J.; Sayle R. A. ZINC20—A Free Ultralarge-Scale Chemical Database for Ligand Discovery. J. Chem. Inf. Model. 2020, 60, 6065–6073. 10.1021/acs.jcim.0c00675. 33118813 PMC8284596

[ref61] O’Boyle N. M.; Banck M.; James C. A.; Morley C.; Vandermeersch T.; Hutchison G. R. Open Babel: An Open Chemical Toolbox. J. Cheminformatics 2011, 3, 33 10.1186/1758-2946-3-33. PMC3198950 21982300

[ref62] Rappe A. K.; Casewit C. J.; Colwell K. S.; Goddard W. A.; Skiff W. M. UFF, a full periodic table force field for molecular mechanics and molecular dynamics simulations. J. Am. Chem. Soc. 1992, 114, 10024–10035. 10.1021/ja00051a040.

[ref63] Koes D. R.; Baumgartner M. P.; Camacho C. J. Lessons Learned in Empirical Scoring with Smina from the CSAR 2011 Benchmarking Exercise. J. Chem. Inf. Model. 2013, 53, 1893–1904. 10.1021/ci300604z. 23379370 PMC3726561

[ref64] Rogers D.; Hahn M. Extended-Connectivity Fingerprints. J. Chem. Inf. Model. 2010, 50, 742–754. 10.1021/ci100050t. 20426451

[ref65] Bajusz D.; Rácz A.; Héberger K. Why Is Tanimoto Index an Appropriate Choice for Fingerprint-Based Similarity Calculations?. J. Cheminformatics 2015, 7, 20 10.1186/s13321-015-0069-3. PMC4456712 26052348

[ref66] Ma C.; Hu Y.; Townsend J. A.; Lagarias P. I.; Marty M. T.; Kolocouris A.; Wang J. Ebselen, Disulfiram, Carmofur, PX-12, Tideglusib, and Shikonin Are Nonspecific Promiscuous SARS-CoV-2 Main Protease Inhibitors. ACS Pharmacol. Transl. Sci. 2020, 3, 1265–1277. 10.1021/acsptsci.0c00130. 33330841 PMC7571300

[ref67] Lee H.; Torres J.; Truong L.; Chaudhuri R.; Mittal A.; Johnson M. E. Reducing Agents Affect Inhibitory Activities of Compounds: Results from Multiple Drug Targets. Anal. Biochem. 2012, 423 (1), 46–53. 10.1016/j.ab.2012.01.006. 22310499 PMC3299889

[ref68] Ihssen J.; Faccio G.; Yao C.; Sirec T.; Spitz U. Fluorogenic in Vitro Activity Assay for the Main Protease Mpro from SARS-CoV-2 and Its Adaptation to the Identification of Inhibitors. STAR Protoc. 2021, 2, 100793 10.1016/j.xpro.2021.100793. 34423318 PMC8367757

[ref69] Hung H.-C.; Ke Y.-Y.; Huang S. Y.; Huang P.-N.; Kung Y.-A.; Chang T.-Y.; Yen K.-J.; Peng T.-T.; Chang S.-E.; Huang C.-T.; Tsai Y.-R.; Wu S.-H.; Lee S.-J.; Lin J.-H.; Liu B.-S.; Sung W.-C.; Shih S.-R.; Chen C.-T.; Hsu J. T.-A. Discovery of M Protease Inhibitors Encoded by SARS-CoV-2. Antimicrob. Agents Chemother. 2020, 64, 64 10.1128/AAC.00872-20. PMC7449189 32669265

[ref70] Lindahl; Abraham; Hess; van der Spoel GROMACS 2021 Source Code, 2021.

[ref71] Jo S.; Kim T.; Iyer V. G.; Im W. CHARMM-GUI: A Web-Based Graphical User Interface for CHARMM. J. Comput. Chem. 2008, 29, 1859–1865. 10.1002/jcc.20945. 18351591

[ref72] Tuccinardi T. What Is the Current Value of MM/PBSA and MM/GBSA Methods in Drug Discovery?. Expert Opin. Drug Discovery 2021, 16 (11), 1233–1237. 10.1080/17460441.2021.1942836. 34165011

[ref73] Tyagi R.; Singh A.; Chaudhary K. K.; Yadav M. K. Chapter 17 - Pharmacophore Modeling and Its Applications. In Bioinformatics; Singh D. B.; Pathak R. K., Eds.; Academic Press, 2022; 269–289.

[ref74] Kralj S.; Jukič M.; Bren U. Commercial SARS-CoV-2 Targeted, Protease Inhibitor Focused and Protein–Protein Interaction Inhibitor Focused Molecular Libraries for Virtual Screening and Drug Design. Int. J. Mol. Sci. 2022, 23, 393 10.3390/ijms23010393. PMC8745317 35008818

[ref75] Zev S.; Raz K.; Schwartz R.; Tarabeh R.; Gupta P. K.; Major D. T. Benchmarking the Ability of Common Docking Programs to Correctly Reproduce and Score Binding Modes in SARS-CoV-2 Protease Mpro. J. Chem. Inf. Model. 2021, 61, 2957–2966. 10.1021/acs.jcim.1c00263. 34047191

[ref76] Macip G.; Garcia-Segura P.; Mestres-Truyol J.; Saldivar-Espinoza B.; Ojeda-Montes M. J.; Gimeno A.; Cereto-Massagué A.; Garcia-Vallvé S.; Pujadas G. Haste Makes Waste: A Critical Review of Docking-Based Virtual Screening in Drug Repurposing for SARS-CoV-2 Main Protease (M-pro) Inhibition. Med. Res. Rev. 2022, 42, 744–769. 10.1002/med.21862. 34697818 PMC8662214

[ref77] Good A. C.; Cho S.-J.; Mason J. S. Descriptors You Can Count on? Normalized and Filtered Pharmacophore Descriptors for Virtual Screening. J. Comput. Aided Mol. Des. 2004, 18 (523), 8211527 10.1007/s10822-004-4065-3. 15729851

[ref78] Oerlemans R.; Ruiz-Moreno A. J.; Cong Y.; Kumar N. D.; Velasco-Velazquez M. A.; Neochoritis C. G.; Smith J.; Reggiori F.; Groves M. R.; Dömling A. Repurposing the HCV NS3–4A Protease Drug Boceprevir as COVID-19 Therapeutics. RSC Med. Chem. 2021, 12, 370–379. 10.1039/D0MD00367K. PMC8130630 34041486

[ref79] Schaller D.; Šribar D.; Noonan T.; Deng L.; Nguyen T. N.; Pach S.; Machalz D.; Bermudez M.; Wolber G. Next Generation 3D Pharmacophore Modeling. WIREs Comput. Mol. Sci. 2020, 10, e1468 10.1002/wcms.1468.

[ref80] Hopkins A. L.; Groom C. R.; Alex A. Ligand Efficiency: A Useful Metric for Lead Selection. Drug Discovery Today 2004, 9, 430–431. 10.1016/S1359-6446(04)03069-7. 15109945

[ref81] Raj V.; Lee J.-H.; Shim J.-J.; Lee J. Antiviral Activities of 4H-Chromen-4-One Scaffold-Containing Flavonoids against SARS–CoV–2 Using Computational and in Vitro Approaches. J. Mol. Liq. 2022, 353, 118775 10.1016/j.molliq.2022.118775. 35194277 PMC8849861

[ref82] Gupta Y.; Kumar S.; Zak S. E.; Jones K. A.; Upadhyay C.; Sharma N.; Azizi S.-A.; Kathayat R. S.; Poonam; Herbert A. S.; Durvasula R.; Dickinson B. C.; Dye J. M.; Rathi B.; Kempaiah P. Antiviral Evaluation of Hydroxyethylamine Analogs: Inhibitors of SARS-CoV-2 Main Protease (3CLpro), a Virtual Screening and Simulation Approach. Bioorg. Med. Chem. 2021, 47, 116393 10.1016/j.bmc.2021.116393. 34509862 PMC8416325

[ref83] Santos L. H.; Kronenberger T.; Almeida R. G.; Silva E. B.; Rocha R. E. O.; Oliveira J. C.; Barreto L. V.; Skinner D.; Fajtová P.; Giardini M. A.; Woodworth B.; Bardine C.; Lourenço A. L.; Craik C. S.; Poso A.; Podust L. M.; McKerrow J. H.; Siqueira-Neto J. L.; O’Donoghue A. J.; da Silva Júnior E. N.; Ferreira R. S. Structure-Based Identification of Naphthoquinones and Derivatives as Novel Inhibitors of Main Protease Mpro and Papain-like Protease PLpro of SARS-CoV-2. J. Chem. Inf. Model. 2022, 62, 6553–6573. 10.1021/acs.jcim.2c00693. 35960688

[ref84] Wang J.; Jiang Y.; Wu Y.; Yu H.; Wang Z.; Ma Y. Pharmacophore-Based Virtual Screening of Potential SARS-CoV-2 Main Protease Inhibitors from the Library of Natural Products. Nat. Prod. Commun. 2022, 17, 1934578X221143635 10.1177/1934578X221143635.

[ref85] Gupta A.; Rani C.; Pant P.; Vijayan V.; Vikram N.; Kaur P.; Singh T. P.; Sharma S.; Sharma P. Structure-Based Virtual Screening and Biochemical Validation to Discover a Potential Inhibitor of the SARS-CoV-2 Main Protease. ACS Omega 2020, 5, 33151–33161. 10.1021/acsomega.0c04808. 33398250 PMC7754785

[ref86] Kneller D. W.; Galanie S.; Phillips G.; O’Neill H. M.; Coates L.; Kovalevsky A. Malleability of the SARS-CoV-2 3CL Mpro Active-Site Cavity Facilitates Binding of Clinical Antivirals. Structure 2020, 28 (12), 1313–1320.e3. 10.1016/j.str.2020.10.007. 33152262 PMC7584437

[ref87] Yoshino R.; Yasuo N.; Sekijima M. Identification of Key Interactions between SARS-CoV-2 Main Protease and Inhibitor Drug Candidates. Sci. Rep. 2020, 10, 12493 10.1038/s41598-020-69337-9. 32719454 PMC7385649

[ref88] Stoddard S. V.; Stoddard S. D.; Oelkers B. K.; Fitts K.; Whalum K.; Whalum K.; Hemphill A. D.; Manikonda J.; Martinez L. M.; Riley E. G.; Roof C. M.; Sarwar N.; Thomas D. M.; Ulmer E.; Wallace F. E.; Pandey P.; Roy S. Optimization Rules for SARS-CoV-2 Mpro Antivirals: Ensemble Docking and Exploration of the Coronavirus Protease Active Site. Viruses 2020, 12, 942 10.3390/v12090942. 32859008 PMC7552026

[ref89] Lee J. T.; Yang Q.; Gribenko A.; Perrin B. S.; Zhu Y.; Cardin R.; Liberator P. A.; Anderson A. S.; Hao L. Genetic Surveillance of SARS-CoV-2 Mpro Reveals High Sequence and Structural Conservation Prior to the Introduction of Protease Inhibitor Paxlovid. mBio 2022, 13, e00869-22 10.1128/mbio.00869-22. 35862764 PMC9426535

[ref90] Ip J. D.; Chu A. W.-H.; Chan W.-M.; Leung R. C.-Y.; Abdullah S. M. U.; Sun Y.; To K. K.-W. Global Prevalence of SARS-CoV-2 3CL Protease Mutations Associated with Nirmatrelvir or Ensitrelvir Resistance. EBioMedicine 2023, 91, 104559 10.1016/j.ebiom.2023.104559. 37060743 PMC10101811

