# Supplementary material for: Consensus Pharmacophore Strategy For Identifying Novel SARS-Cov-2 Mpro Inhibitors from Large Chemical Libraries
Source: J Chem Inf Model. 2024 Mar 12;64(6):1984–95. doi: 10.1021/acs.jcim.3c01439 (PMC10966741; doi:10.1021/acs.jcim.3c01439)
Supplement: Supplementary file 1 — ci3c01439_si_001.pdf [file ci3c01439_si_001.pdf]

A Consensus Pharmacophore Strategy For Identifying Novel Sars-Cov-2 M^{pro} Inhibitors From Large Chemical Libraries

Angel J. Ruiz-Moreno[a *], Raziel Cedillo-González[a,b,c], Luis Cordova-Bahena[a,d], Zhiqiang An[e] José L. Medina-Franco[c], and Marco A. Velasco-Velázquez[a,e *]

[a] School of Medicine, Universidad Nacional Autónoma de México, Mexico City 04510, Mexico

[b] Graduate Program in Biochemical Sciences, Universidad Nacional Autónoma de México, Mexico City 04510, Mexico

[c] DIFACQUIM research group, School of Chemistry, Universidad Nacional Autónoma de México, Mexico City 04510, Mexico

[d] Consejo Nacional de Humanidades, Ciencias y Tecnología, Mexico City 03940, Mexico

[e] Texas Therapeutics Institute, Brown Foundation Institute of Molecular Medicine, University of Texas Health Science Center, Houston, Texas 77030, USA

* angel_ruiz_m@comunidad.unam.mx or marcovelasco@unam.mx

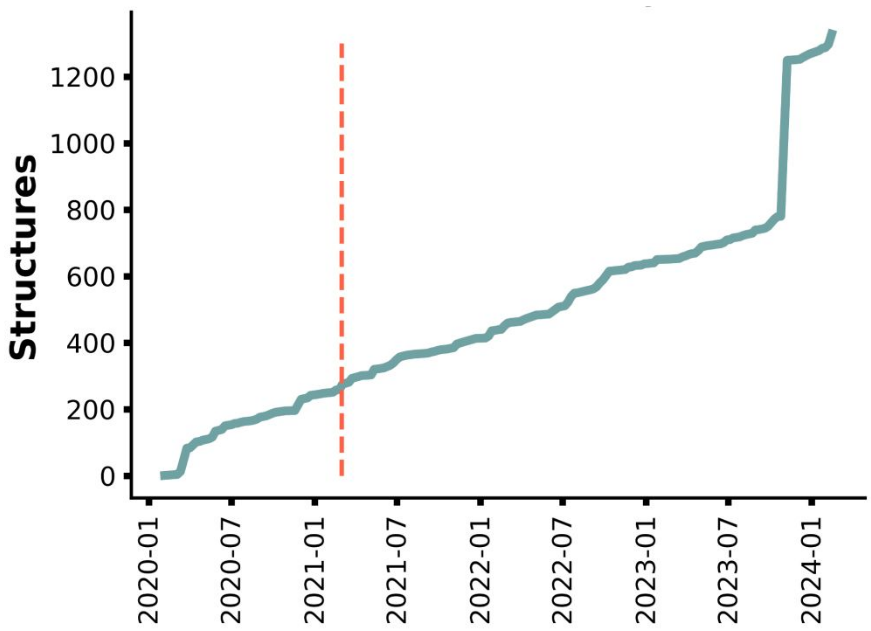

Figure S1. Number of Mpro structures available in PDB over time. Dotted red line correspond to the date in which the structures employed for our study were collected.

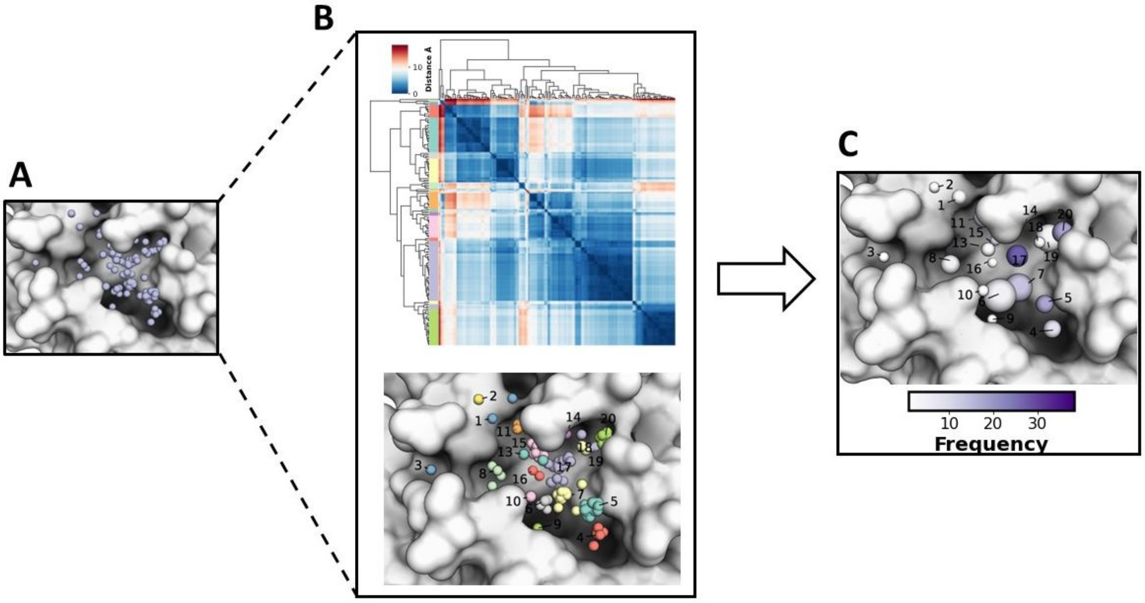

Figure S2. Identification of consensus pharmacophoric points for hydrogen donors.

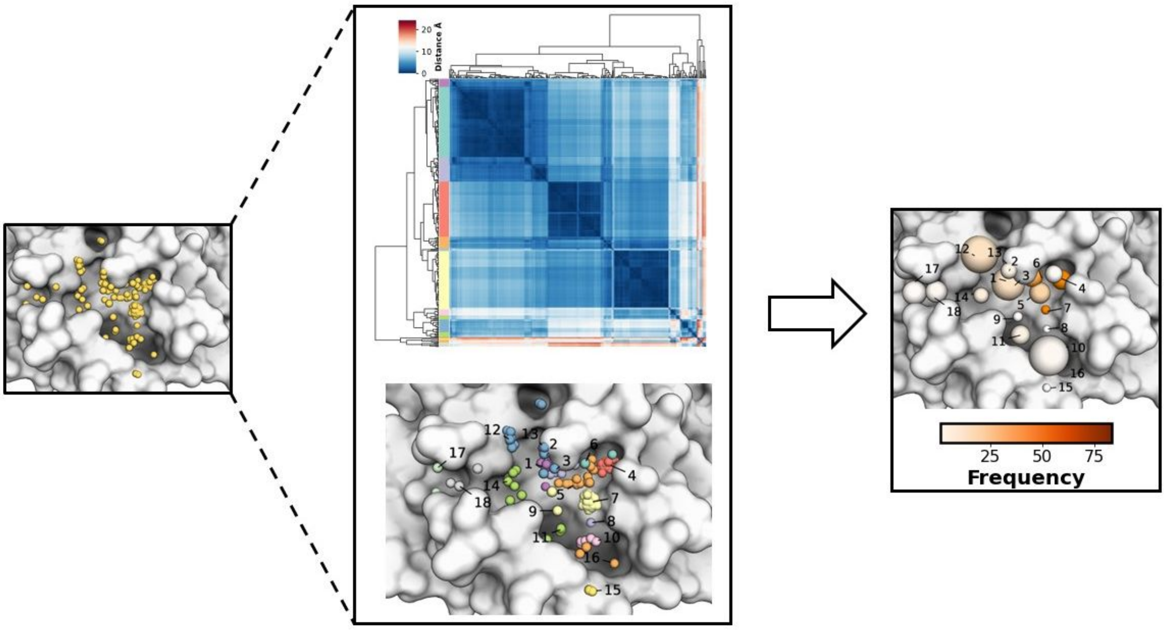

Figure S3. Identification of consensus pharmacophoric points for hydrogen acceptors.

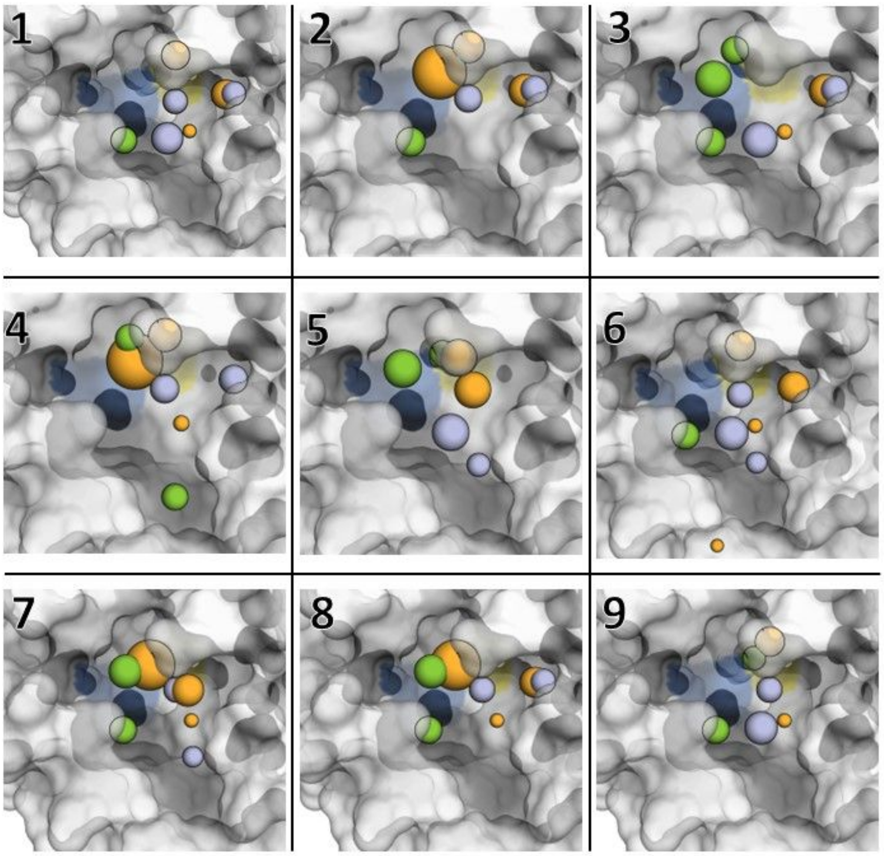

Figure S4. Pharmacophoric submodels generated from the consensus pharmacophore.

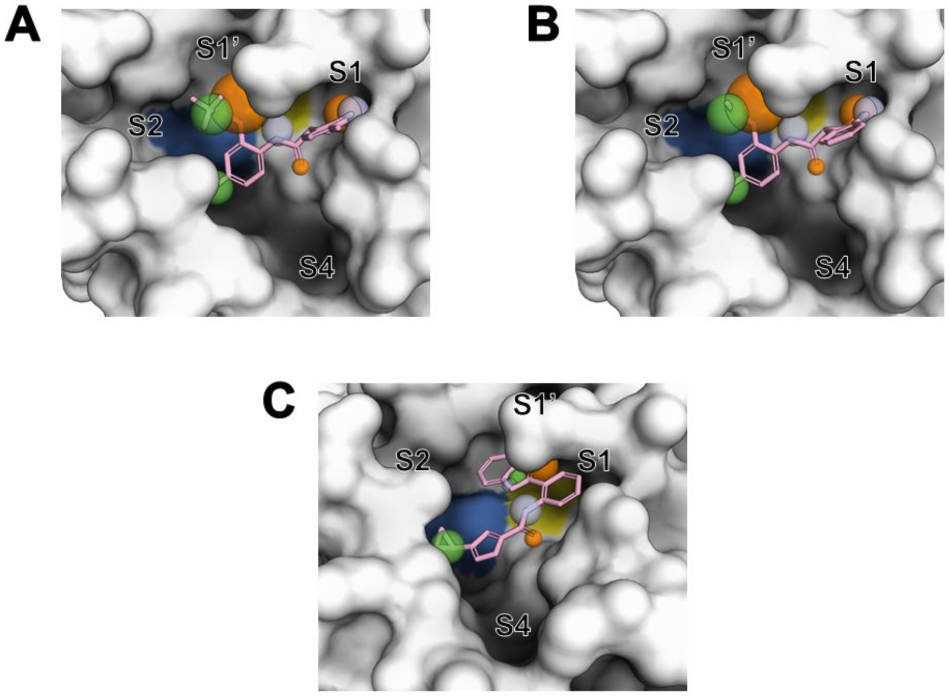

Figure S5. Pharmacophore matching of the candidate compounds 1 (**A**), 4 (**B**), and 5 (**C**) within M^{pro} catalytic site subpockets. The spheres represent the consensus pharmacophoric descriptors used to select the compounds. Hydrophobic elements are green, hydrogen bond acceptors are orange, and hydrogen bond donors are blue.

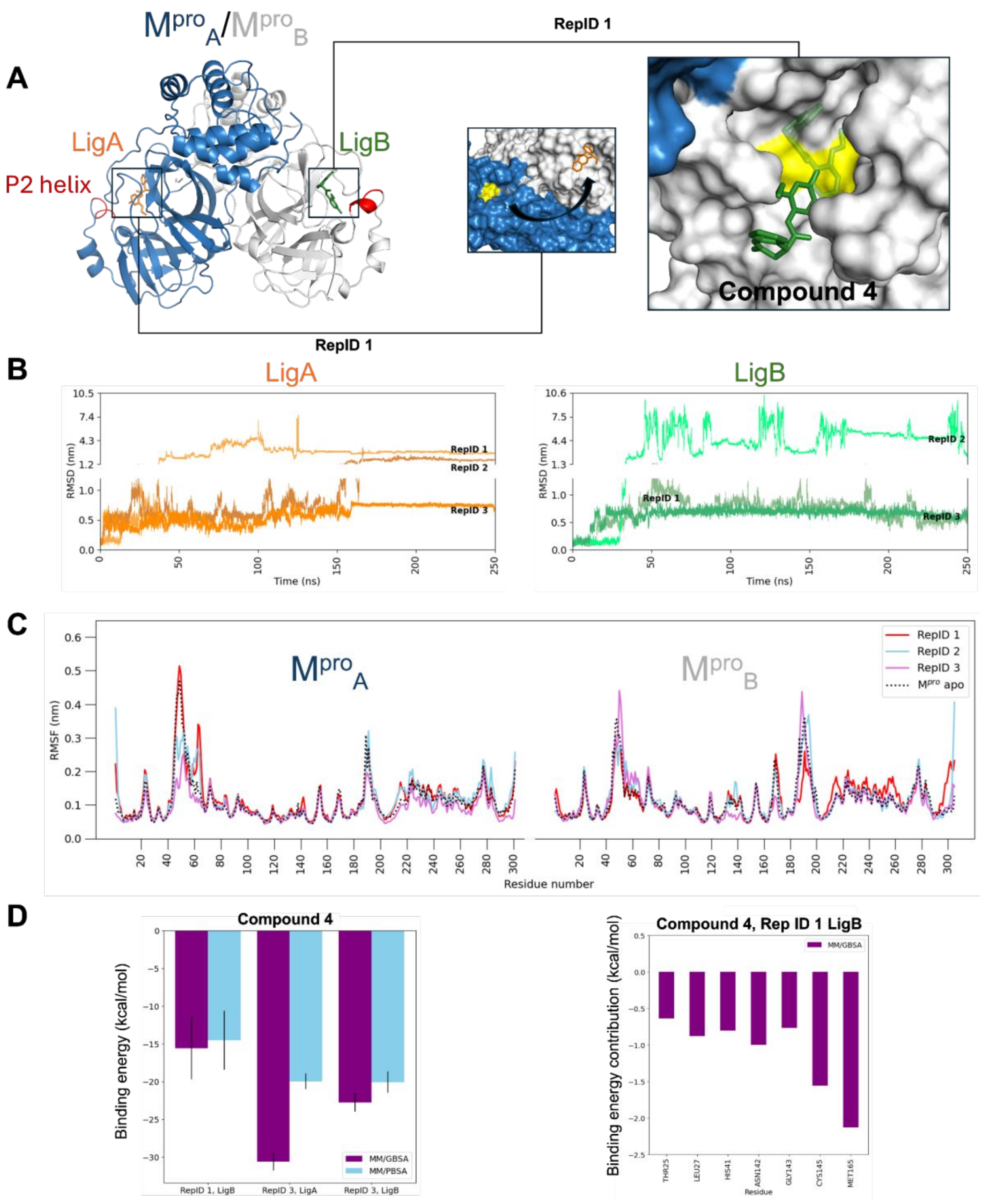

Figure S6. Analysis of molecular dynamics simulations for complex M^{pro}-compound 4. **A**) M^{pro} protomers are shown in blue (M^{pro}_A) and white (M^{pro}_B). Each catalytic cavity had one ligand (LigA and LigB, shown as stick models in orange and green, respectively). The insets display the initial conformation (transparent sticks) and a representative conformer from MD simulation (solid sticks). The catalytic dyad is depicted in yellow, and the P2 helix is highlighted in red for both monomers. **B**) RMSD graphs for compound 4 (LigA and LigB) in three different replicates of MD simulation. **C**) RMSF graphs for alpha-carbons of M^{pro} protomers A and B with ligands in three different replicates of MD simulation. The Mpro dimer without ligand (apo) is presented for comparison. **D**) Binding energy calculated for the ligands that remained bound, using the molecular mechanics generalized Born surface area (MM/GBSA) and Poisson–Boltzmann surface area (MM/PBSA) methods. **E**) Contribution by residue to binding energy for LigB in RepID 1 by MM/GBSA.

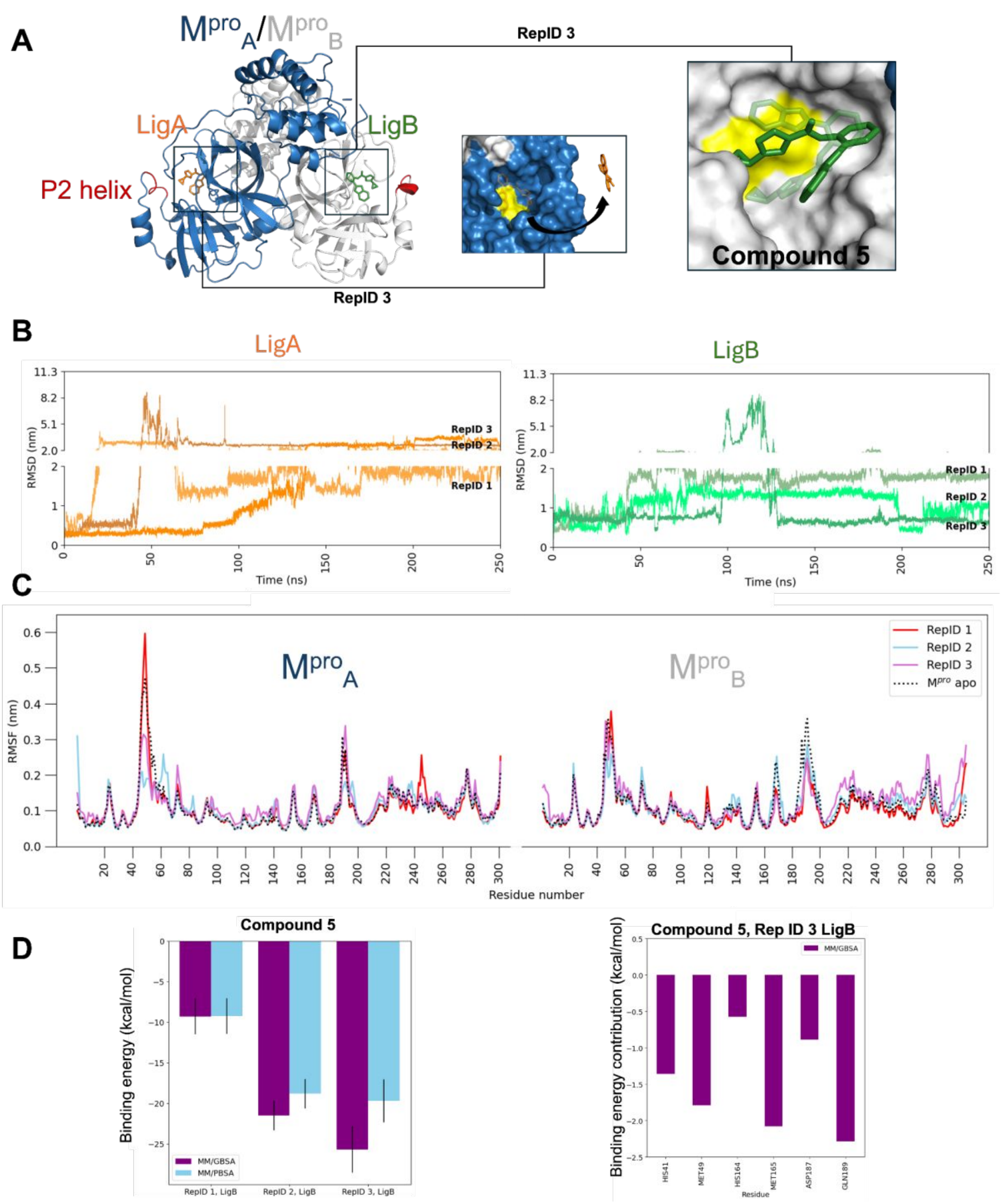

Figure S7. Analysis of molecular dynamics simulations for complex M^{pro}-compound 5. **A**) M^{pro} protomers are shown in blue (M$^{pro}{}_{A}$) and white (M$^{pro}{}_{B}$). Each catalytic cavity had one ligand (LigA and LigB, shown as stick models in orange and green, respectively). The insets display the initial conformation (transparent sticks) and a representative conformer from MD simulation (solid sticks). The catalytic dyad is depicted in yellow, and the P2 helix is highlighted in red for both monomers. **B**) RMSD graphs for compound 5 (LigA and LigB) in three different replicates of MD simulation. **C**) RMSF graphs for alpha-carbons of M^{pro} protomers A and B with ligands in three different replicates of MD simulation. The Mpro dimer without ligand (apo) is presented for comparison. **D**) Binding energy calculated for the ligands that remained bound, using the molecular mechanics generalized Born surface area (MM/GBSA) and Poisson–Boltzmann surface area (MM/PBSA) methods. **E**) Contribution by residue to binding energy for LigB in RepID 3 by MM/GBSA.

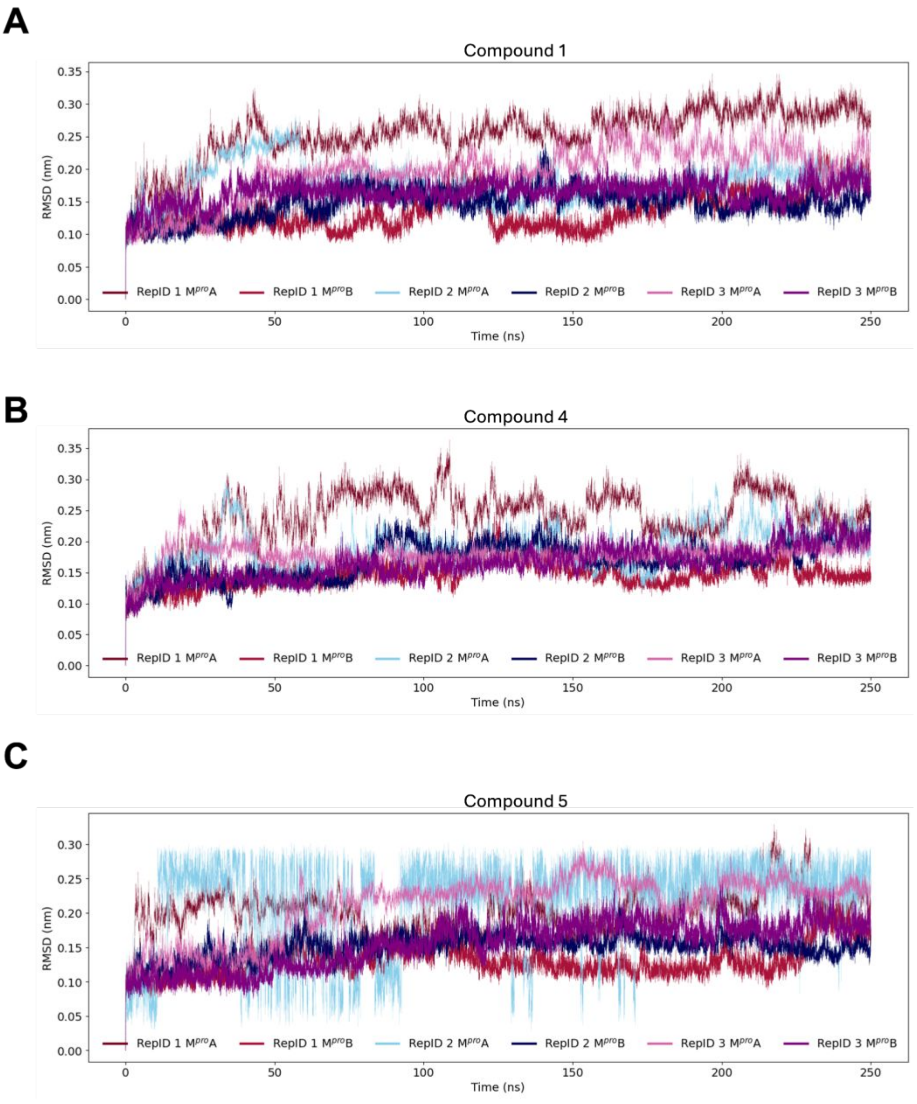

Figure S8. RMSD of M^{pro} alpha-carbons from molecular dynamics simulations with compounds 1 (**A**), 4 (**B**), and 5 (**C**).
